# Development of participatory intervention to improve safety and enjoyment in sports events based on Participatory Action-Oriented Training

**DOI:** 10.3389/fspor.2026.1979235

**Published:** 2026-10-23

**Authors:** Yasuyuki Yamada, Yuriko Takeuchi, Toyoki Nakao, Motoko Kai, Takuya Tanaka, Wei Guo, Sora Watabe, Saki Hosoi, Kazutaka Kogi, Toru Yoshikawa, Etsuko Yoshikawa, Takashi Toriizuka, Atsushi Kubota, Koya Suzuki, Ko Yamanaka, Motoki Mizuno, Takeshi Hibi, Kazuhiro Aoki, Daichi Suzuki, Hidefumi Waki

**Affiliations:** 1 Graduate School of Health and Sports Science, Juntendo University, Inzai, Chiba, Japan; 2 The Ohara Memorial Institute for Science of Labour, Toshima, Tokyo, Japan; 3 Tokyo Occupational Safety and Health Center, Koto, Tokyo, Japan; 4 National Institute of Occupational Safety and Health, Kawasaki, Kanagawa, Japan; 5 Japanese Red Cross College of Nursing, Shibuya, Tokyo, Japan; 6 College of Industrial Technology, Nihon University, Narashino, Chiba, Japan

**Keywords:** Human Factors and Ergonomics, Participatory Action-Oriented Training (PAOT), risk management, science communication, sports events, sport management

## Abstract

**Introduction:**

Sports events are leisure activities that contribute to public well-being. To maximize their benefits, large-scale improvements and the continuous accumulation of small, low-cost, and immediately actionable improvements are needed. However, systematic approaches for the improvement of safety and enjoyment in sports events remain limited. Hence, this study developed and evaluated the Sports Event Improvement for Safe, Move, Info, Link, and Environment (SMILE) program based on Participatory Action-Oriented Training (PAOT).

**Methods:**

The SMILE program was developed using a research and development framework and evaluated through field research and questionnaire. Field research covered professional football, basketball, and rugby games in Japan. The program involved 294 participants in Day 1 fieldwork and 243 in Day 2 workshops. Output evaluation assessed good practices, improvement proposals based on the program design principle of balancing safety and enjoyment, club feedback, and participant satisfaction. Outcome evaluation examined pre–post changes in engagement with sports clubs, spectating, supporting, and participating in sports, and PAOT.

**Results:**

Participants collected 2,951 photographs of good practices and proposed 174 improvements. Output evaluation demonstrated high participant satisfaction and positive club feedback. Outcome evaluation showed significant pre–post increases in engagement with sports and PAOT. A 30-item action checklist based on photographs was developed to support the program.

**Discussion:**

The SMILE program was developed as a participatory approach to the sustained improvement of safety and enjoyment in sports events. It promotes the refinement and sharing of good practices, the accumulation of small, low-cost, and immediately actionable improvements, and value co-creation among stakeholders.

## 1 Introduction

Spectating is a popular leisure activity requiring extensive public support. It increases interest in sports (1), promotes physical activity and health (2), and enhances life satisfaction (3). Moreover, it contributes to subjective happiness and overall well-being (4, 5). However, unsatisfactory safety and service quality during sporting events may diminish these benefits. For instance, spectators at professional sporting events may be dissatisfied if the match is not exciting, players underperform, or the atmosphere is unpleasant (6). Poor hospitality, limited facility access, or uncomfortable venue conditions may further reduce satisfaction (6). Poor security can lead to critical incidents (7). Poor risk management can contribute to spectator accidents (8), spectator violence (9, 10), noise hazards (7), infectious disease transmission (11, 12), heat-related illnesses (13), terrorism (14), and unexpected disasters (15). Therefore, organizers and clubs must pursue improvements to maximize the benefits and mitigate risks.

Research in sports management has identified key contributors to game satisfaction, including winning outcomes and athlete performance, termed “core elements” or “core products” (6, 16, 17). In contrast, service satisfaction is influenced by “extended elements” or “ancillary services,” including visual effects, music, programs, facilities, concessions, and safety measures. To enhance service satisfaction, sports management researchers have improved service quality to further improve these externally controllable, extended factors (6, 16–18). In particular, safety-related service-quality improvements in sports events should balance risk reduction with enjoyment, as excessive safety measures can inadvertently diminish the excitement of live sports (8). Hence, organizers and clubs must pursue improvements in these extended elements to maintain a balance between safety and enjoyment.

Numerous sports organizations have already implemented measures to elevate spectator safety and service quality at events (19, 20). For instance, elite clubs have leveraged substantial management resources to develop expertise regarding large-scale events (21), while minor clubs adapt creatively to facilities and services despite their limited resources (16, 22). Additionally, previous studies pertaining to risk management in sports events typically have reported large-scale interventions that require advanced technologies, substantial financial investment, and long-term planning, particularly in the context of mega-sports events (23–25). However, not all clubs and events can operate with substantial financial resources. Therefore, organizers and clubs need to learn from good practices already implemented in the field and accumulate small, low-cost, and immediately actionable improvements.

Furthermore, recent studies in sport entertainment have highlighted the importance of “value co-creation,” in which the emotional and symbolic value of spectating experiences is co-created by multiple stakeholders, including clubs and fans (26, 27). However, systematic approaches to facilitate stakeholder collaboration in sports spectating remain limited. Hence, organizers and clubs must pursue participatory and co-creative approaches that enable multiple stakeholders, including spectators, athletes, staff, and local communities, to collaborate and continuously improve sports events.

Therefore, this study explores Participatory Action-Oriented Training (PAOT), a methodology used in the fields of Human Factors and Ergonomics (HFE). PAOT was developed by the International Labour Organization (ILO) to improve workplace safety, health, and productivity (28–30). It encompasses simple procedures for advancing the Plan–Do–Check–Act (PDCA) cycle by focusing on good practices using practical toolkits (31–33). It encompasses three interlinked steps: learning from good local practices using photographs, videos, and action checklists (31, 32); group work involving self-help actions (31) in which stakeholders collaboratively co-create improvement measures, encouraging active participation (31, 34); and obtaining immediate results with follow-up (31). PAOT encourages low-cost and simple improvements that can be implemented immediately and whose effects can be readily assessed (31, 33, 35). Although PAOT was originally developed to improve workplace environments in small enterprises, its versatility has led to its widespread application in various settings (28, 36). Customized PAOT packages have been developed for small enterprises, agricultural and construction sites, healthcare facilities, and educational institutions (28–30, 34, 37–41). Additionally, PAOT promotes continuous self-help voluntary actions (28) and sustainable improvements (33), and thus exhibits strong potential to improve safety and enjoyment in sports events.

Accordingly, this study aims to develop and evaluate a novel approach—the Sports Event Improvement for Safe, Move, Info, Link, and Environment (SMILE) program—designed to improve safety and enjoyment in sports events.

## 2 Materials and methods

### 2.1 Study design

To develop the SMILE program, this study adopted a research and development (R&D) approach comprising five stages: conceptual design, pilot implementation, output evaluation, outcome evaluation, and program refinement. A mixed-methods approach combining a field study and pre–post questionnaire surveys was used to evaluate the program.

### 2.2 Participants

Eligible participants were citizens residing in Chiba Prefecture, Japan, where the hometowns of the three professional sports clubs involved in this study are located, and students attending universities in Chiba Prefecture. Participants were recruited on the basis that they would attend both days of the two-day SMILE program. Figure 1 shows the participant flow from registration through the final matched pre–post analysis sample.

**Figure 1:**
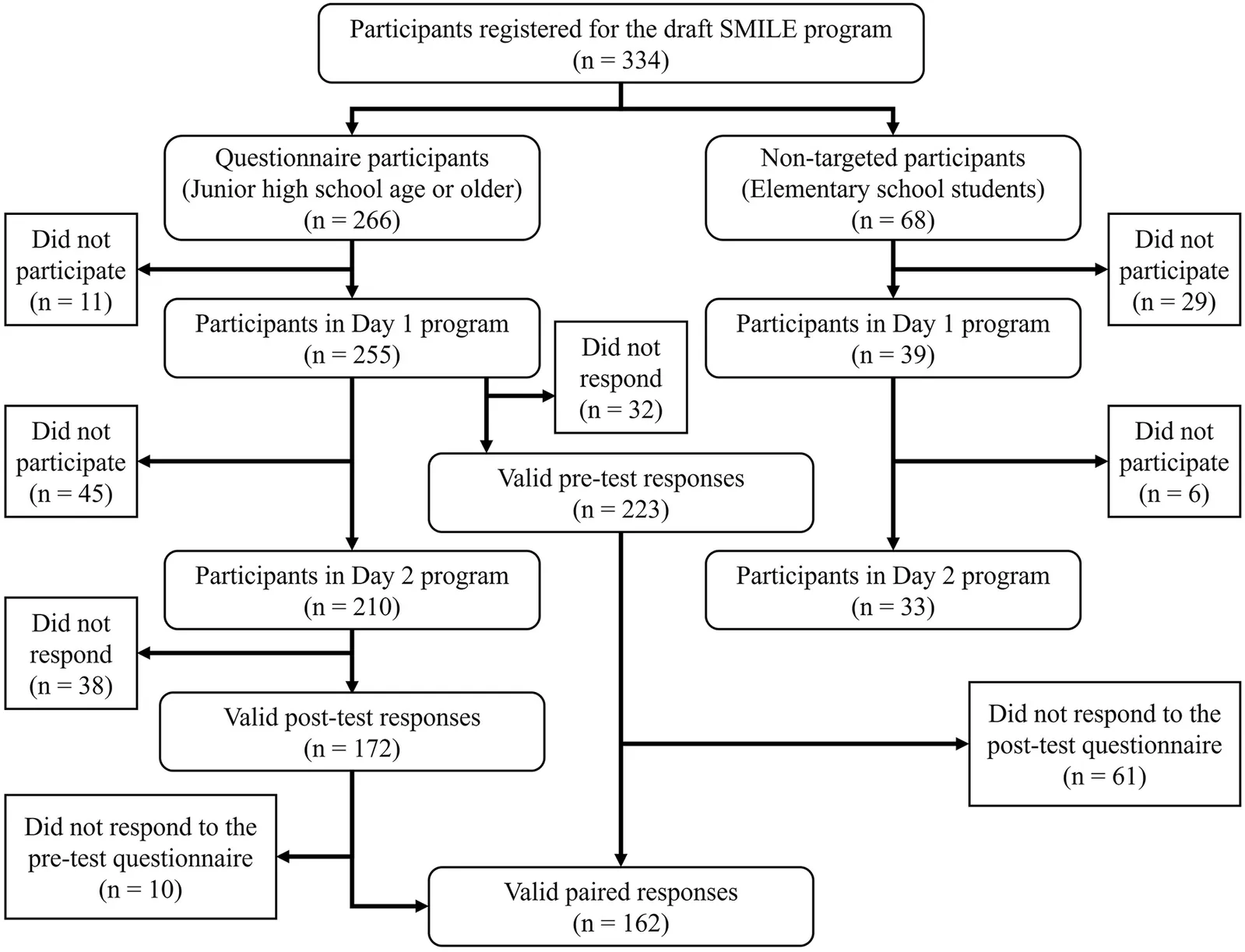
Participant flow diagram for the draft SMILE program.

On the first day, 294 participants attended the fieldwork session (Table 1). The pre-test questionnaire targeted 255 participants of junior high school age or older, excluding 39 elementary school students. Of these, 223 provided valid responses (response rate: 87.5%; mean age: 25.35 ± 13.12 years). On the second day, 243 participants attended the workshop session (Table 1). The post-test questionnaire targeted 210 participants of junior high school age or older, excluding 33 elementary school students. Of these, 172 provided valid responses (response rate: 81.9%; mean age: 27.03 ± 11.71 years). A total of 162 participants provided valid responses to both the pre- and post-test questionnaires (mean age: 26.56 ± 11.54 years). Approximately 80% of participants had no previous experience of attending a home game of the professional sports club they selected for the SMILE program (pre-test data: 78.1%; post-test data: 79.7%; matched pre–post data: 78.4%).

**Table 1:** Number of participants in the two-day SMILE program.

Professional sports club	Date of fieldwork	Number of participants (Elem.)	Date of workshop	Number of participants (Elem.)
JEF United (Football)	2022/09/25	31 (8)	2022/10/22	33 (7)
	2022/10/16	61 (12)	2022/10/29	35 (7)
CHIBA JETS (Basketball)	2022/10/02	39 (3)	2022/10/29	69 (7)
	2022/10/15	37 (5)	2022/11/05	34 (4)
	2022/10/23	33 (3)		
Kubota Spears (Rugby)	2023/01/14	46 (4)	2023/01/22	38 (2)
	2023/01/21	47 (4)	2023/01/28	34 (6)
Total		294 (39)		243 (33)

Elem. denotes elementary school students. JEF United denotes JEF United Ichihara Chiba (J. League), CHIBA JETS denotes CHIBA JETS (B. LEAGUE), and Kubota Spears denotes Kubota Spears Funabashi Tokyo Bay (Japan Rugby League One).

Among all participants, 110 participated with their families, representing 42 families. As participation by parents and children could introduce bias during group discussions and decision-making processes, measures were taken to minimize parental influence during the workshop on the second day. Specifically, elementary school students were assigned to groups consisting of only children for the group work and presentations. Junior high and high school students were also assigned to separate groups from their parents or guardians. Because questionnaire completion was optional and pre–post analyses required matched responses from both surveys, the number of valid responses differed across the analyses.

### 2.3 Study setting and collaborating organizations

This study was conducted through a multi-sector collaboration involving Juntendo University; the Japan Sports Agency; three professional sports clubs: JEF United Ichihara Chiba [hereafter JEF United; Japan Professional Football League (J. League)], CHIBA JETS [Japan Professional Basketball League (B.LEAGUE)], and Kubota Spears Funabashi TOKYO-BAY (hereafter Kubota Spears; Japan Rugby League One); local governments (Inzai City, Sakura City, and Shisui Town); the Ohara Memorial Institute for Science of Labour; and participating citizens. Juntendo University planned and managed this study, and the Japan Sports Agency provided financial support. Three professional sports clubs provided fieldwork opportunities and evaluated citizen-submitted improvement proposals. Local governments promoted citizen participation. The Ohara Memorial Institute provided technical support for the PAOT. Citizens voluntarily participated in the SMILE program and contributed photographs depicting good practices in event operations, improvement proposals, and evaluations of program outcomes. Although participation was voluntary, the research budget covered transportation costs and admission fees for the professional sporting events associated with the intervention.

### 2.4 Procedure

The development and evaluation of the SMILE program involved a series of expert meetings with six researchers: five specializing in HFE and occupational safety and health (OSH), and one specialized in sports management and HFE. All six had completed international PAOT programs, had developed PAOT packages, and served as coordinators or trainers in international PAOT workshops, including the Mekong Delta (42) and Japan–Korea sessions (43).

Four of the researchers had previously developed and evaluated PAOT packages (30, 41, 44, 45). This study adopted a five-step procedure based on the R&D framework commonly referenced in previous studies on applied PAOT programs (34, 44, 46, 47): (1) conceptual design, (2) pilot implementation, (3) output evaluation, (4) outcome evaluation, and (5) program refinement (Table 2).

**Table 2:** Five-stage process used to develop the SMILE program based on an R&D framework.

Stage	Purpose	Main activities	Outputs
1. Conceptual design	Define the framework of the SMILE program	Expert meetings Literature review	SMILE concept
2. Pilot implementation	Develop a draft program and checklist and test the intervention	Fieldwork Workshops	Draft program Draft checklist
3. Output evaluation	Assess program feasibility and implementation outputs	Photo analysis Proposal analysis Post-program survey	Good-practice photos Improvement proposals Program satisfaction
4. Outcome evaluation	Assess participant-level engagement outcomes	Pre–post survey	Engagement outcomes
5. Program refinement	Refine the draft program and checklist	Program revision Checklist revision	Refined program Refined checklist

#### 2.4.1 Conceptual design procedure (stage 1)

In Stage 1, the HFE experts defined the scope, principles, and implementation requirements of the SMILE program through a review of previous PAOT studies and field observations at professional sporting events. The concept of the SMILE program was developed based on the definition of HFE established by the IEA (48). The principles of the SMILE program were defined based on previous PAOT literature. The five SMILE domains (Safe, Move, Info, Link, and Environment) were examined and operationally defined through expert field observations and meetings. In addition, conceptual definitions were established for the “action checklist” used as a practical toolkit to support improvement activities and for “good practices” collected by participants during fieldwork. The action checklist is a toolkit used to support improvement activities, including learning from good local practices, identifying significant risks and effective measures, planning locally feasible improvements, implementing prioritized measures, recording achievements, and reviewing performance to sustain stepwise progress (33).

#### 2.4.2 Pilot implementation procedure (stage 2)

In Stage 2, a draft program and checklist were developed through a series of expert meetings and subsequently implemented in collaboration with professional sports clubs. The draft checklist was designed as a practical support tool for PAOT implementation and was not intended for psychometric evaluation. Its practical relevance was examined through field observations by HFE experts and inductive content analysis of photographs depicting good practices collected during preliminary observations at professional sporting events.

The draft program was constructed through expert consensus in three steps (1): designing content based on the core principles of the applied PAOT programs; (2) creating a program timetable with reference to the existing PAOT package guidelines; and (3) testing and refining the draft based on field implementation.

The draft checklist was developed with reference to the “Ergonomic Checkpoints” published by the ILO and International Ergonomics Association (IEA) (49) following these steps: (1) agreeing on the main domains requiring immediate improvements based on local good practices; (2) selecting 30–50 checkpoint titles, with several assigned to each area; (3) testing the draft checklist and formulating a locally adjusted version, with a focus on low-cost improvements; and (4) supplementing the checklist with a brochure containing corresponding pages to serve as reference material for users. Steps (1) and (2) were implemented as part of the pilot process. Step (3) was performed during program refinement (Stage 5), whereas Step (4) remains a task for future research.

Three of the six HFE experts described above agreed on the main checklist domains and developed checklist items. The experts examined the domains of good practice using photographs collected during preliminary observations at professional sporting events in baseball, football, rugby, and basketball that differed from those included in this study. The numbers and names of the good-practice domains used in the program were determined with reference to previous PAOT packages. Subsequently, six photographs of good practices were selected for each of the five SMILE domains, and checklist item descriptions corresponding to each photograph were developed, resulting in a draft checklist consisting of 30 items (Figure 2). The draft checklist was used prior to the fieldwork to facilitate the participants’ understanding of good practices.

**Figure 2:**
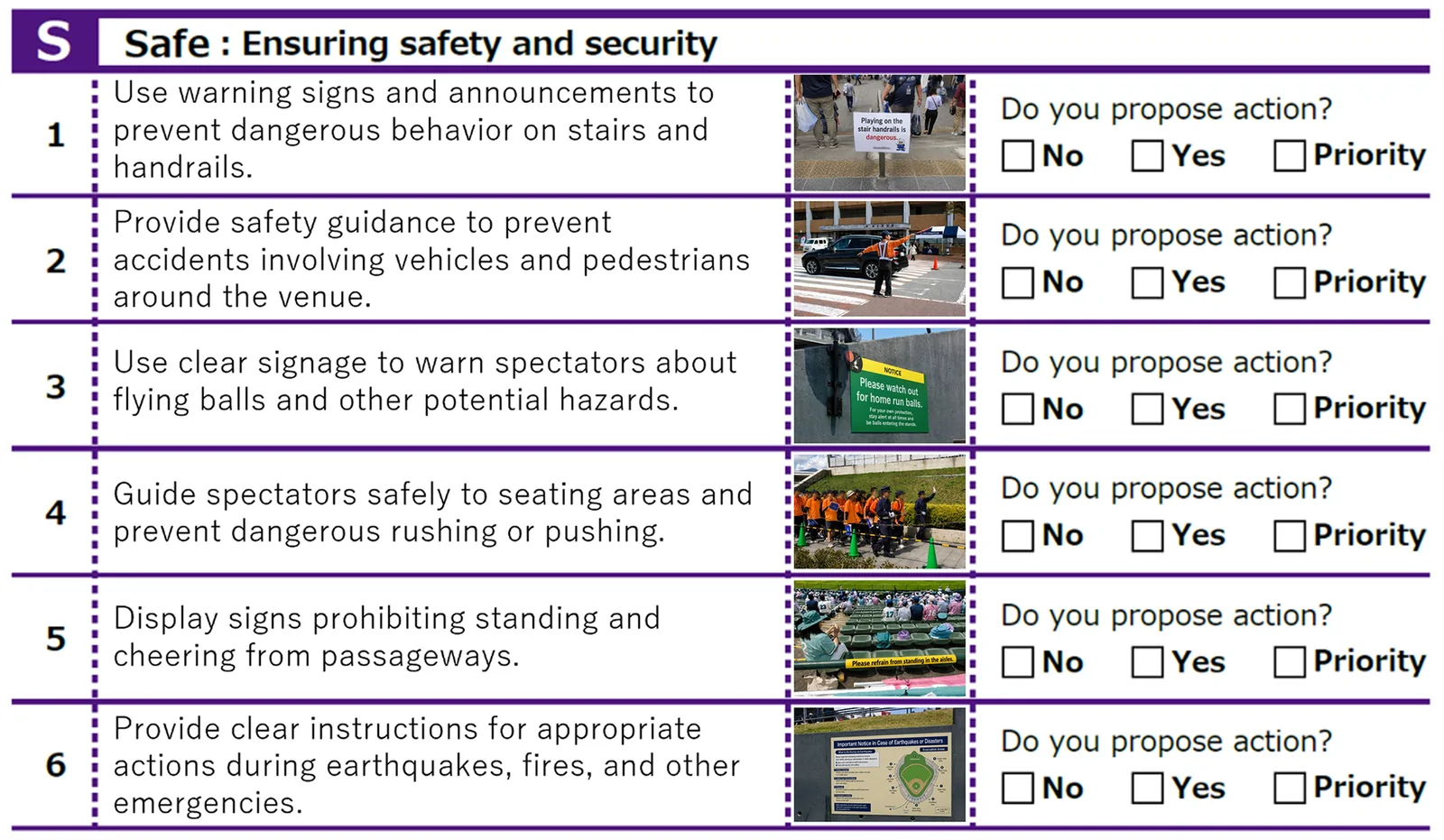
Six checkpoints from the “Safe” domain of the draft 30-item SMILE action checklist. Japanese text appearing in the original photographs was translated and digitally modified into English using AI-assisted image editing, while preserving the original content and layout.

The pilot implementation was conducted across three sports: football (J. League) and rugby (Japan Rugby League One), both held in stadiums, and basketball (B. LEAGUE), held in arenas. The targeted matches included two home games of JEF United at Fukuda Denshi Arena, three games of CHIBA JETS at Funabashi Arena, and two games of Kubota Spears at the Edogawa City Track and Field Stadium during the 2022–2023 season. Each participant selected one of the seven professional home games across the three leagues and documented good practices while watching the game.　Participants attended the workshop corresponding to the club at which they had conducted the fieldwork and were assigned to groups according to the same SMILE domain as in the fieldwork. Participants were assigned to groups by the facilitators. To minimize potential bias associated with parent–child participation, elementary school students were assigned to groups consisting only of children and participated in a simplified version of the program. Junior and senior high school students were assigned to groups separate from their parents or guardians. Where possible, citizens and university students were mixed across groups to avoid excessive concentrations of specific generations.

The participants were recruited through volunteer sampling. Eligible individuals included residents of Chiba Prefecture, where the three clubs were based, and students enrolled in educational institutions in the same region. Recruitment was conducted through posters featuring QR codes for online applications displayed by three local governments and one university in the Chiba Prefecture. Two of the six HFE experts described above facilitated the pilot implementation workshops.

#### 2.4.3 Output evaluation procedure (stage 3)

In Stage 3, the outputs of the improvement activities conducted during the pilot implementation were evaluated. Evaluation indicators included photographs documenting good practices, improvement proposals submitted to professional sports clubs, feedback received from the clubs, and participant satisfaction with the program.

For good practices, the total number of photographs collected was used as a quantitative indicator. In addition, based on the design principle of the SMILE program, two HFE experts classified the good-practice photographs within each SMILE domain into those that considered the enjoyment of sports events and those that represented good practices but were not directly related to the enjoyment of sports events. The two experts conducted the classification through discussion, and any differences in judgment were resolved by consensus. Representative examples of good practices that considered the enjoyment of sports events were selected for each club and SMILE domain.

For improvement proposals, the total number of proposals generated by participants was used as a quantitative indicator. In addition, based on the design principle of the SMILE program, two HFE experts classified the improvement proposals within each SMILE domain into those that considered the enjoyment of sports events and those that represented necessary improvements but were not directly related to the enjoyment of sports events. The two experts conducted the classification through discussion, and any differences in judgment were resolved by consensus. Representative examples of improvement proposals that considered the enjoyment of sports events were selected for each club and SMILE domain.

Thus, to evaluate the balance between safety and enjoyment, a design principle of the SMILE program, as a program output, consideration of enjoyment was assessed in the qualitative evaluation of good practices and improvement proposals. Here, enjoyment was evaluated as a characteristic of the program outputs rather than as an individual-level outcome among program participants.

Separately, participant satisfaction with the SMILE program was assessed using the 172 valid responses to the post-test questionnaire. The method used to assess satisfaction was based on a previous study of sports event satisfaction (50), in which satisfaction with individual aspects of a sports event was assessed using single-item ratings on a 5-point Likert scale ranging from “very dissatisfied” to “very satisfied.” The participants were asked to rate their satisfaction with each component of the SMILE program using the question: “How satisfied were you with the following components of the SMILE program?” The six items assessed were (1) fieldwork, (2) game observations, (3) Introductory session, (4) group work, (5) presentations, and (6) the overall program. Responses were recorded on a 5-point Likert scale ranging from 1 (“very dissatisfied”) to 5 (“very satisfied”).

#### 2.4.4 Outcome evaluation procedure (stage 4)

In Stage 4, an outcome evaluation was conducted to examine participant-level changes in engagement using a pre–post comparison design in which the same questionnaire was administered before and after participation in the draft SMILE program in Stage 2. This outcome evaluation was not intended to assess changes in event safety or enjoyment *per se*, but rather to examine participant-level changes in engagement before and after participation in the SMILE program. Engagement was assessed across five domains: sports club, spectating sports, supporting sports, participating in sports, and participating in PAOT. The outcome evaluation was based on the 162 participants who provided valid responses to both the pre- and post-test questionnaires.

To assess changes in engagement with the sports club, this study used the items of the Psychological Continuum Model (PCM) developed by Kunkel et al. (51). This scale assesses participants’ psychological connection (involvement) with a professional sports club across three dimensions: pleasure [3 items, e.g., Watching the (LEAGUE/TEAM) is one of the most satisfying things I do], sign [3 items, e.g., Watching the (LEAGUE/TEAM) says a lot about who I am], and centrality [3 items, e.g., I find a lot of my life organized around following the (LEAGUE/TEAM)]. In the original scale, the target was expressed as “[LEAGUE/TEAM]”; in this study, the actual name of the professional sports club at which each participant conducted the fieldwork was inserted in place of “[LEAGUE/TEAM].” The PCM items were translated into Japanese and back-translated into English to confirm consistency with the original items. Responses were recorded on a 7-point Likert scale ranging from 1 (“strongly disagree”) to 7 (“strongly agree”).

To evaluate changes in engagement in the four domains of spectating sports, supporting sports, participating in sports, and participating in PAOT using a common conceptual framework, this study developed original items based on the AEIOU model of science communication (52). The items were developed by one expert in science communication and one expert in ergonomics, who reviewed previous studies on science communication evaluation and reached consensus on the items after considering the definitions of each AEIOU dimension and the forms of engagement expected through participation in the SMILE program. The AEIOU framework evaluates personal responses across five dimensions—Awareness, Enjoyment, Interest, Opinion formation, and Understanding—and has been applied to evaluate science communication experiences (52, 53). The SEC-AEIOU questionnaire (53), a previously developed measure, was developed using a sample of university students and uses open-ended responses for Opinion formation and true-or-false questions for Understanding. These methods were burdensome to administer within a limited time to participants including junior and senior high school students, and it was also difficult to adjust their difficulty to allow consistent assessment across the broad age range from junior and senior high school students to adults. Therefore, in the present study, the items were developed as practical indicators, with each AEIOU dimension assessed using a single item to facilitate responses from participants, including junior and senior high school students, while allowing engagement to be assessed across multiple dimensions. The same 5-point response format used to assess Awareness, Enjoyment, and Interest in the SEC-AEIOU was adopted. The primary outcome indicators were the pre–post changes in the five AEIOU dimensions within each of the four engagement domains.

For each engagement domain, participants responded to items corresponding to the five AEIOU dimensions. For example, in the “spectating sports” domain, participants were asked, “When you watch sports games, to what extent do the following statements apply to you?” The five items were: (1) “I become more aware” (Awareness), (2) “I find it enjoyable” (Enjoyment), (3) “I am interested in it” (Interest), (4) “I have my own opinions and ideas” (Opinion formation), and (5) “I have sufficient knowledge and understanding” (Understanding). Responses were recorded on a 5-point Likert scale ranging from 1 (“strongly disagree”) to 5 (“strongly agree”), based on the AEIOU-based outcome evaluation (53). The questionnaires are provided in Supplementary Material 1.

#### 2.4.5 Program refinement procedure (stage 5)

In Stage 5, the draft program and checklist were revised by the six HFE experts based on the outcomes of the pilot implementation. Expert meetings involving facilitators of pilot sessions focused on improving the productivity and operational efficiency of the program, including timetable adjustments.

The refined version of the action checklist was developed using good-practice photographs collected during the pilot implementation in Stage 2. The checklist was developed from the photographic data following the inductive content analysis process proposed by Elo and Kyngäs (54), namely, open coding, grouping, categorization, and abstraction (Table 3). The two HFE experts did not conduct the coding independently; rather, they jointly reviewed the photographs and conducted open coding, grouping, categorization, and abstraction through discussion. When their judgments differed during the analytical process, they discussed the issue until consensus was reached. Therefore, inter-rater agreement was not calculated. Through this process, open codes were generated from the photographic data, codes with similar meanings were grouped, and these groups were integrated into categories based on common meanings within each SMILE domain and subsequently abstracted. Representative good-practice photographs were then selected for each category, and corresponding checklist items were developed. Furthermore, illustrations for each checklist item were created based on the selected representative good-practice photographs to help users visualize the intended good practices. The draft checklist was used during the pilot implementation in Stage 2 to facilitate the collection of good practices, and the refined checklist was developed based on the good-practice photographs collected in this study. The checklist format adopted the “No,” “Yes,” and “Priority” response options in accordance with the ergonomic checkpoint guidelines published by the ILO and IEA (49).

**Table 3:** Inductive content analysis procedures based on Elo and Kyngäs (54).

Stage	Purpose
1. Open coding	Good-practice photographs from each SMILE domain were used as data, and the characteristics and meanings contained in the photographs were identified.
2. Grouping	Photographs with similar characteristics and meanings were grouped.
3. Categorization	Conceptually distinct categories were extracted based on recurring characteristics and meanings observed in the photographs.
4. Abstraction	Representative good-practice photographs were selected for each category and corresponding checklist items were developed. Illustrations corresponding to each checklist item were also developed.

### 2.5 Statistical analysis

The web-based questionnaires used in this study could only be submitted after all items had been completed; therefore, there were no item-level missing data in the collected questionnaire data, and no imputation was performed.

First, to examine potential selection bias due to attrition in the matched pre–post analysis, baseline characteristics were compared between participants with matched pre–post data and those who completed the pre-test questionnaire but did not provide corresponding post-test data. Age was compared using an independent-samples t-test, whereas participant category (non-student adults, university students, and junior/senior high school students), sex (male and female), previous viewing experience (whether participants had previously attended a home game of the professional sports club they selected for the SMILE program: yes or no), and intervention club (JEF United, CHIBA JETS, and Kubota Spears) were compared using chi-square tests.

Second, to evaluate participant satisfaction with the program, descriptive statistics were calculated for satisfaction scores for each program component. In addition, to examine whether satisfaction differed by participant category, satisfaction scores were compared across participant categories using one-way analysis of variance (ANOVA). These analyses were based on data from participants who submitted the post-test questionnaire.

Third, to examine whether engagement scores changed before and after participation in the draft SMILE program, differences between pre-test and post-test scores were analyzed using paired t-tests. The pre–post analyses were conducted using complete data from participants who submitted both the pre-test and post-test questionnaires. Furthermore, to account for multiple testing, the Holm adjustment was applied to the pre–post tests of engagement with the sports club, spectating sports, supporting sports, participating in sports, and participating in PAOT.

Finally, analysis of covariance (ANCOVA) was conducted to examine whether post-test engagement scores varied according to participant characteristics and external factors after adjusting for baseline scores, with the post-test score as the dependent variable, participant category, sex, previous viewing experience, and intervention club as fixed factors, and the corresponding pre-test score as the covariate. Participant category and sex were included as participant characteristics potentially related to engagement with sports, previous viewing experience as a factor potentially related to engagement with the respective club, and intervention club as an event-related factor potentially reflecting differences in sport type and event environment. Furthermore, to account for multiple testing, the Holm adjustment was applied to the tests of the main effects of participant category, sex, previous viewing experience, and intervention club across the engagement outcomes. All statistical analyses were conducted using IBM SPSS Statistics, version 28.0.1.0.

## 3 Results

### 3.1 Results of conceptual design (stage 1)

Based on the definition of HFE proposed by the IEA (48), HFE experts defined the SMILE program as “the scientific discipline concerned with the understanding of interactions among stakeholders involved in sports events and other elements of a system, and the profession that applies theory, principles, data, and methods of PAOT to design to optimize well-being in sports events and overall system performance.” By this definition, stakeholders include spectators, residents, athletes, sponsors, clubs, events, and volunteer staff. Other elements included venue design, event operations, and service delivery. In the context of wellbeing, sports spectating facilitates game satisfaction, service satisfaction, and experiential value, whereas system performance encompasses entertainment, safety, comfort, audience attraction, financial sustainability, and social impact. The SMILE program targeted five thematic areas for intervention, based on observed good practices in professional sporting events and previous PAOT package domains: ensuring safety and security (safe), smooth movement (move), communication and information (info), people-friendly facility maintenance (link), and environmental conservation (environment). In particular, the Link domain emphasizes inclusive support for diverse stakeholders. These domains were operationally defined through expert observations of professional sporting events and their alignment with the existing PAOT frameworks. HFE experts also derived five key approaches for the SMILE program from previous PAOT studies: a participatory approach that encourages multiple stakeholders to collaborate and pursue improvements from diverse perspectives; a positive approach that focuses on identifying and expanding existing good practices in the field; a proposal-oriented approach that avoids abstract criticism and promotes constructive suggestions for improvement; a dialogue-oriented approach that facilitates two-way communication; and a feasibility-oriented approach that favors low-cost, immediately actionable improvements.

The action checklist is a practical toolkit that combines illustrated checkpoints with concise descriptions to support the identification of good practices and areas for improvement during sporting events (55). In previous PAOT packages, action checklists have been used to encourage action-oriented sustainable improvements through participants’ own efforts (37). Based on previous PAOT guidelines (30, 37, 45–47), the checklist was divided into multiple domains with specific checklist items assigned to each domain. The checklist was designed to allow users to select and adapt domains and checklist items according to the local field conditions (49). Accordingly, the action checklist was defined as “a checklist designed to support the recognition of good practices and the creation of practical improvement proposals observed in the context of sports events, with the assumption that it will be localized to fit specific field conditions.”

Finally, “good practices” were operationally defined as “initiatives that are subjectively perceived as favorable by at least one stakeholder at a given point in time,” reflecting the context-dependent nature of what is considered “good” according to differences in goals, timing, location, and stakeholder perspectives in practice.

### 3.2 Results of pilot implementation (stage 2)

The draft SMILE program was designed by HFE experts with extensive PAOT experience (Table 4). The program adopted the key characteristics of PAOT-based packages, including good-practice orientation, serial group work steps, localized toolkits, and workshop-style delivery formats (33, 45). As sports club staff members are typically unavailable during games, the program was structured as a two-day intervention: the first on the game day and the second on a separate day. To reduce barriers to citizen participation, the duration was kept short: four hours on the first day and three hours on the second day. Participants were divided into groups according to their SMILE domains, and each group was responsible for collecting photographs depicting examples of good practices and proposing improvement measures within their assigned domains.

**Table 4:** Schedule of the two-day draft SMILE program.

Time	Day 1	Time	Day 2
00:00	(1) Assembly and registration	00:00	(1) Opening session
00:00–00:30	(2) Orientation	00:00–00:20	(2) Introductory session
	-Explanation of the SMILE program		-Introduction of the sports club
	-Introduction of good practices from		-Explanation of PAOT
	other events using photographs and videos	00:20–01:30	(3) Group work (with breaks)
	-Explanation of the action checklist		-Icebreaker
	-Grouping based on SMILE domains		-Selection of top 3 good practices
00:30–02:00	(3) Fieldwork		-Discussion of further improvement
	-Collection of good practices at the venue	01:30–02:40	(4) Presentations (with breaks)
02:00–04:00	(4) Game observation		-Presentation of top 3 good practices
	-Collection of good practices in game operations		-Presentation of improvement proposals
	-Sharing photographs of good practices		-Feedback from the sports club
04:00	(5) Dismissal (Participants are free to leave after the end of the game)	02:40–03:00	(5) Awards and summary
		03:00	(6) Closing session

SMILE denotes Sports Event Improvement for Safe, Move, Info, Link, and Environment; PAOT denotes Participatory Action-Oriented Training.

The pilot study was conducted in collaboration with three professional sports clubs over seven matches (JEF United, *n* = 2; CHIBA JETS, *n* = 3; Kubota Spears, *n* = 2). A total of 294 individuals participated on the first day (JEF United, *n* = 92; CHIBA JETS, *n* = 109; Kubota Spears, *n* = 93), and 243 participated on the second day (JEF United, *n* = 68; CHIBA JETS, *n* = 103; Kubota Spears, *n* = 72). The draft action checklist developed by the HFE experts based on onsite observations was used to guide the identification of good practices and support proposal development.

### 3.3 Results of output evaluation (stage 3)

The output evaluation examined the quantity and qualitative characteristics of the good-practice photographs and improvement proposals, feedback received from the clubs, and participants’ satisfaction. On the first day, 294 participants conducted fieldwork involving the collection of good-practice photographs and game observations at sports venues. A total of 2,951 good-practice photographs were collected across seven matches (Table 5). To qualitatively evaluate the collected good-practice photographs, they were classified within each SMILE domain according to whether they considered the enjoyment of sports events. As shown in Table 6, good practices that considered the enjoyment of sports events were identified across all clubs and SMILE domains, including “Manners signs with football mascot,” which encouraged appropriate spectator behavior while harmonizing with the event atmosphere, and “Team-colored cable covers,” which minimized visual disruption to the venue environment.

**Table 5:** Number of participants and good-practice photographs collected in day 1 fieldwork.

Sports club	Date of fieldwork	Number of participants (Elem.)	Total number of collected good-practice photographs						
			Safe	Move	Info	Link	Envi.	Others	Total
JEF United (Football)	2022/09/25	31 (8)	47	59	120	49	39	0	314
	2022/10/16	61 (12)	37	131	135	46	71	38	458
CHIBA JETS (Basketball)	2022/10/02	39 (3)	67	156	208	32	44	37	544
	2022/10/15	37 (5)	48	99	89	38	74	3	351
	2022/10/23	33 (3)	32	44	82	73	133	26	390
Kubota Spears (Rugby)	2023/01/14	46 (4)	64	195	166	91	44	16	576
	2023/01/21	47 (4)	65	117	53	77	6	0	318
Total		294 (39)	360	801	853	406	411	120	2,951

Elem. denotes elementary school students and Envi. denotes environment. JEF United denotes JEF United Ichihara Chiba (J. League), CHIBA JETS denotes CHIBA JETS (B. LEAGUE), and Kubota Spears denotes Kubota Spears Funabashi Tokyo Bay (Japan Rugby League One).

**Table 6:** Examples of collected good practices that considered the enjoyment of sports events in the SMILE program.

SMILE	JEF United (Football)	CHIBA JETS (Basketball)	Kubota Spears (Rugby)
Safe	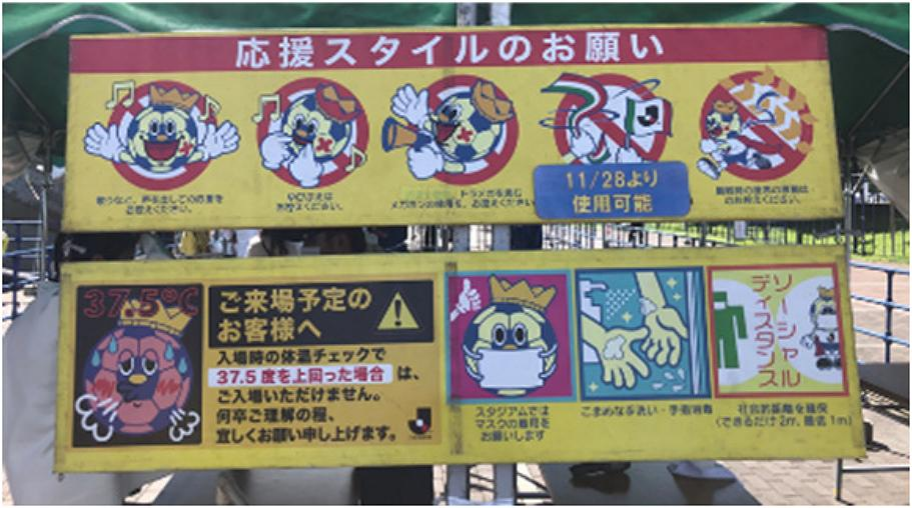 Manners signs with football mascot	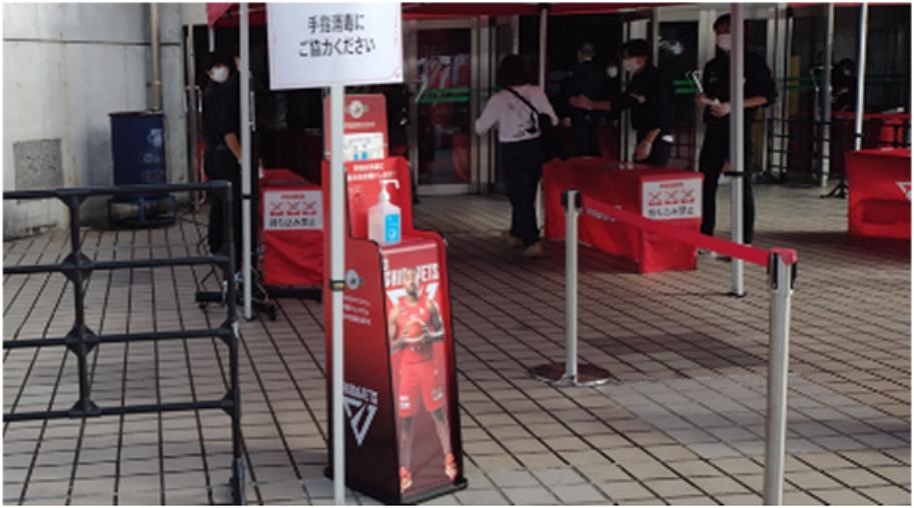 Sanitizer stand with player image	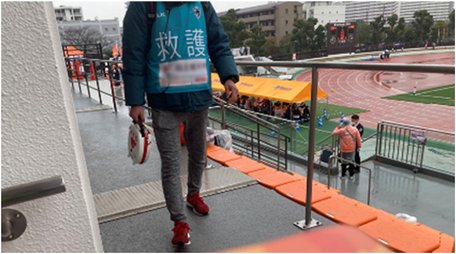 Mobile staff with AED
Move	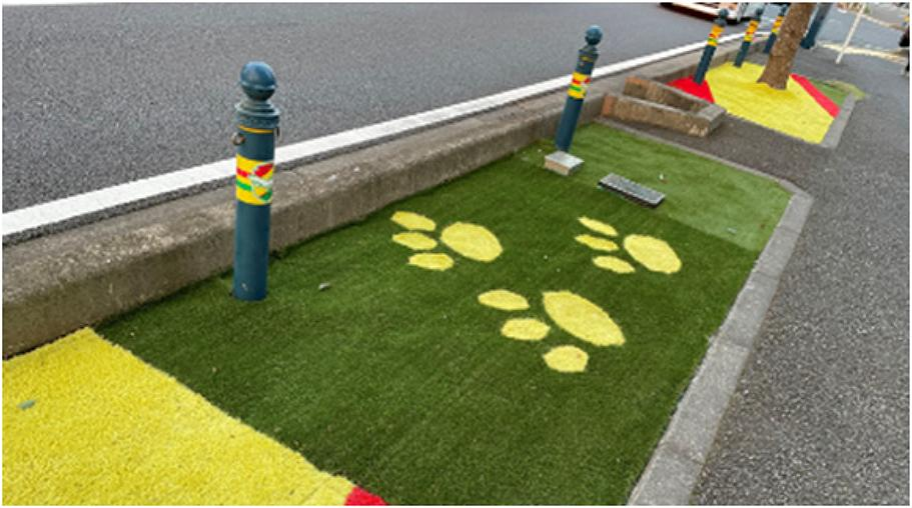 Team mascot-themed stadium signs	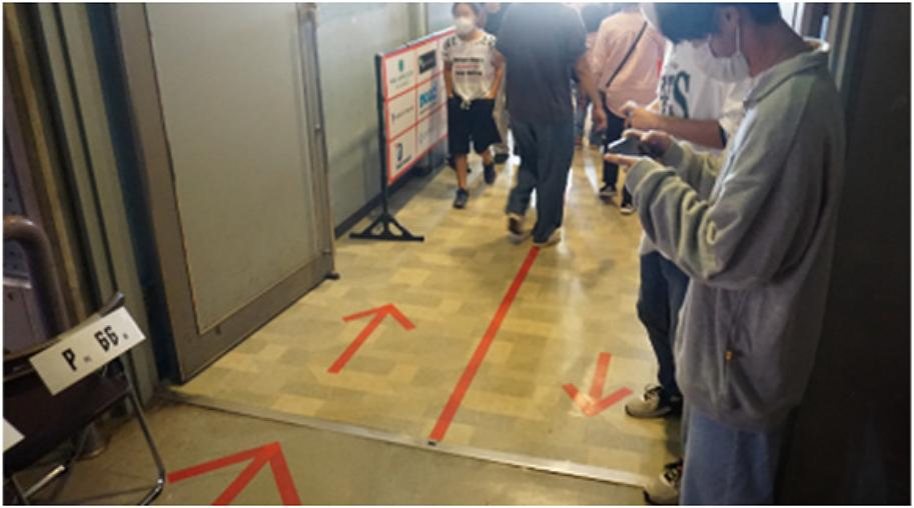 Team-colored floor lines for flow	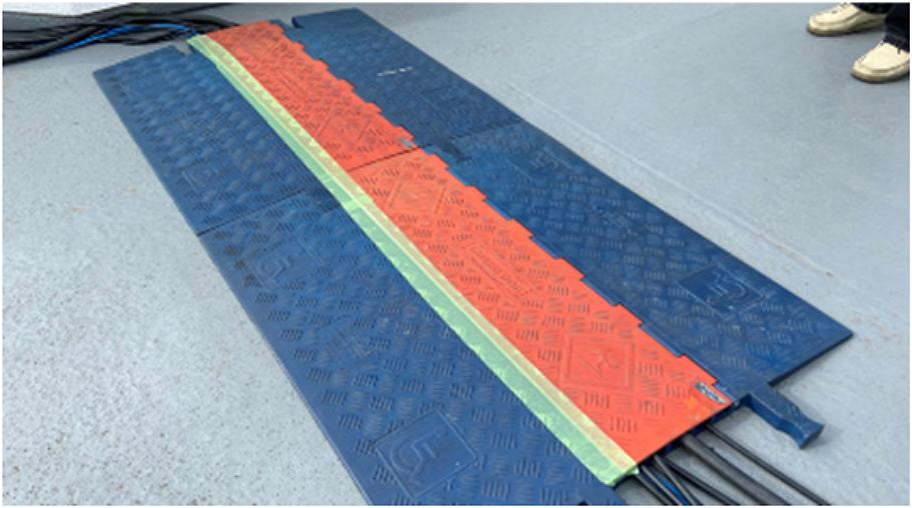 Team-colored cable covers
Info	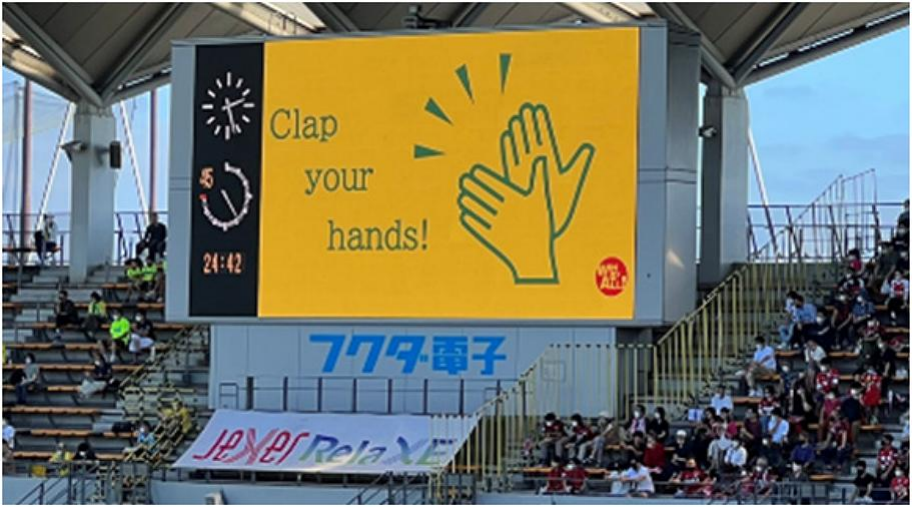 Cheer guide for first-timers	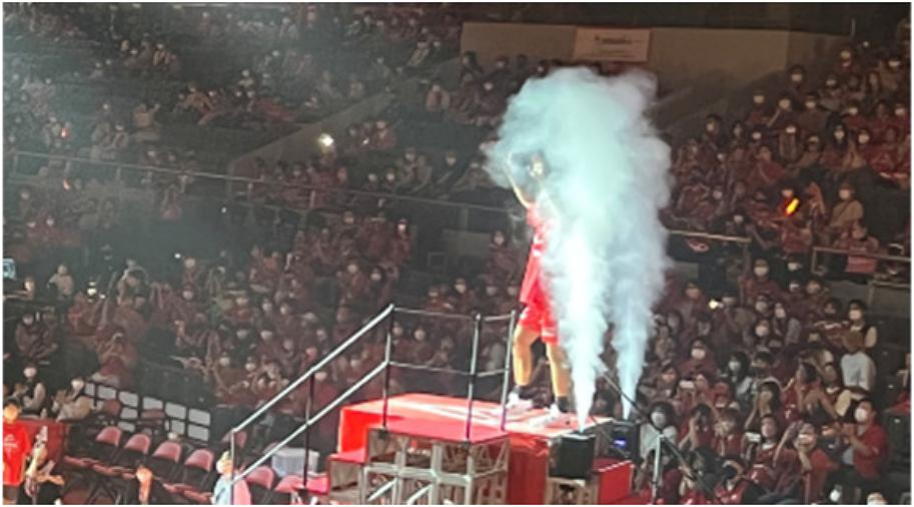 Podium to enhance visibility of player entrance	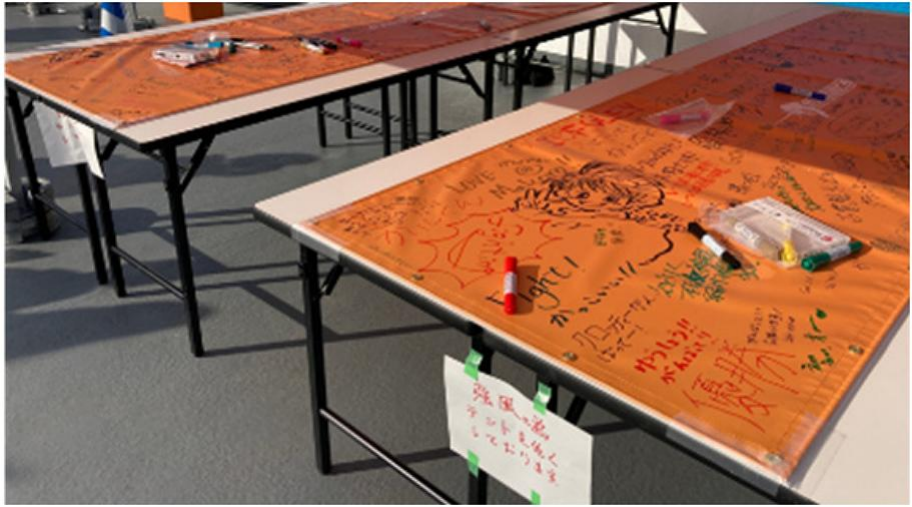 Message banner for players
Link	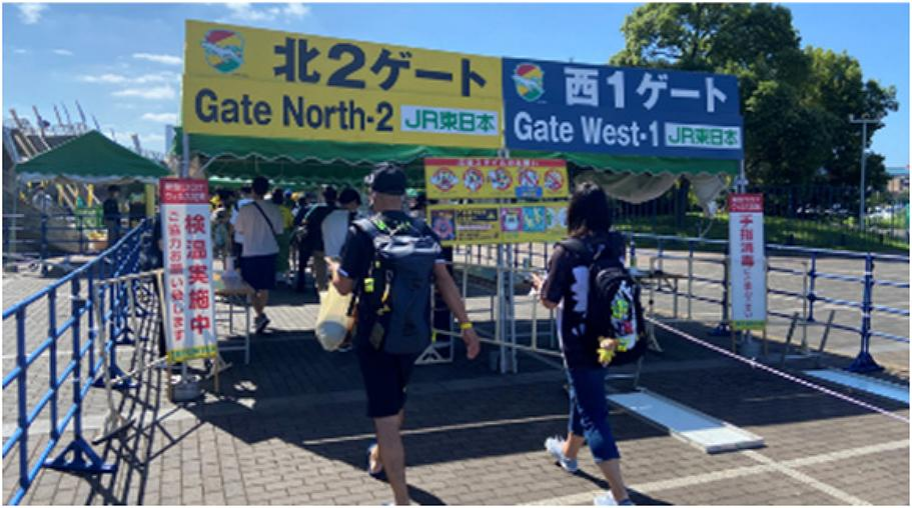 Multilingual signs for visitors	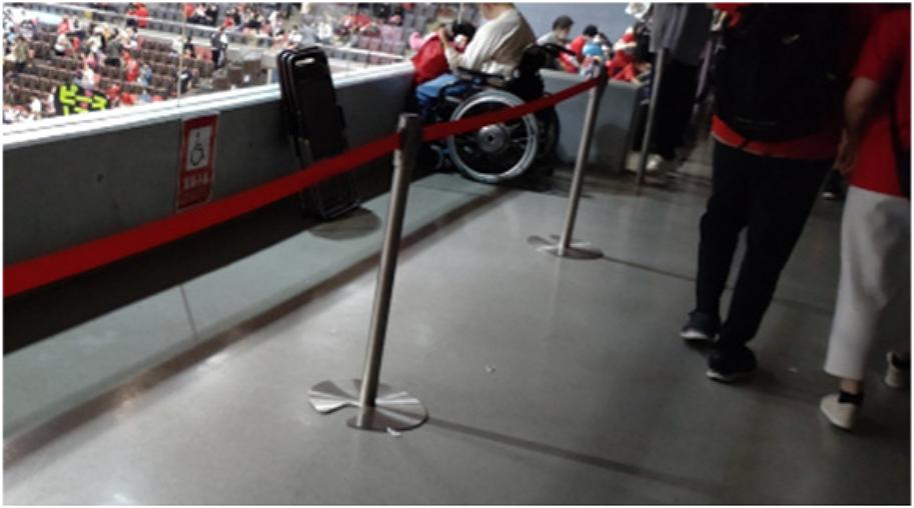 Designated wheelchair cheer zone	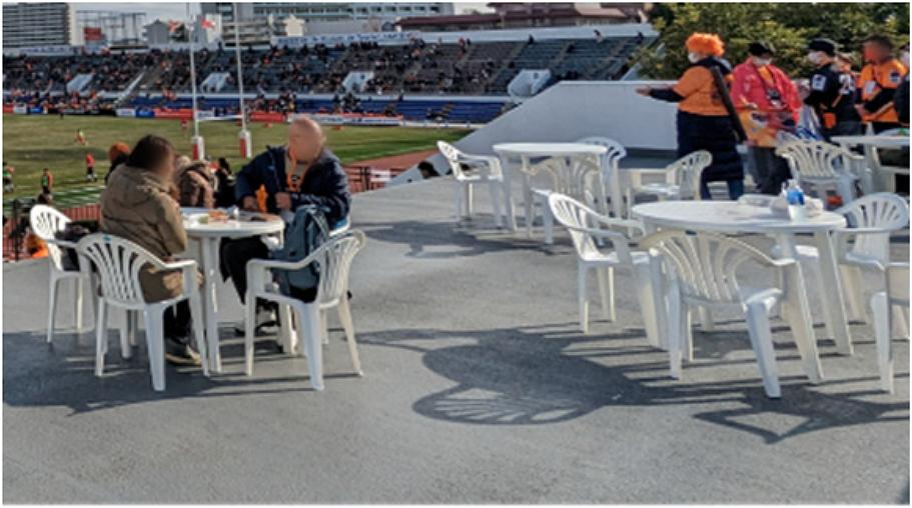 Rest area for tired spectators
Envi.	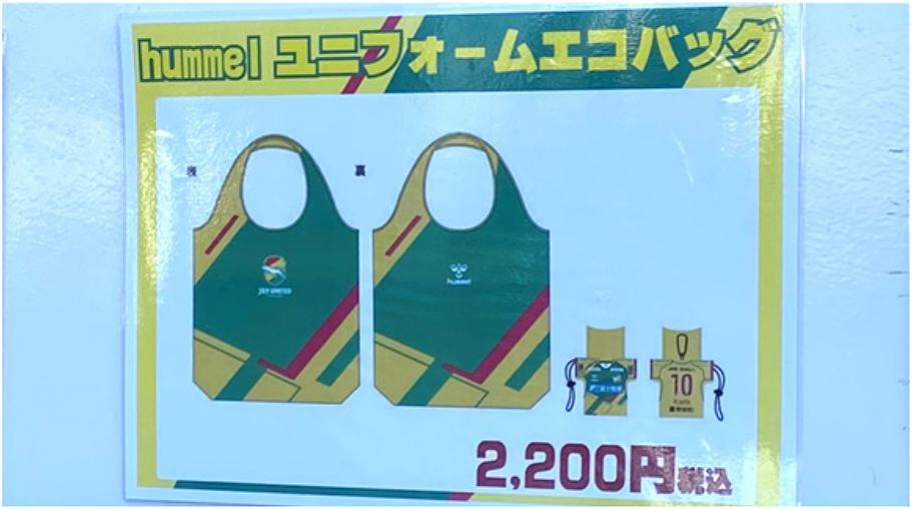 Eco-bag with uniform design	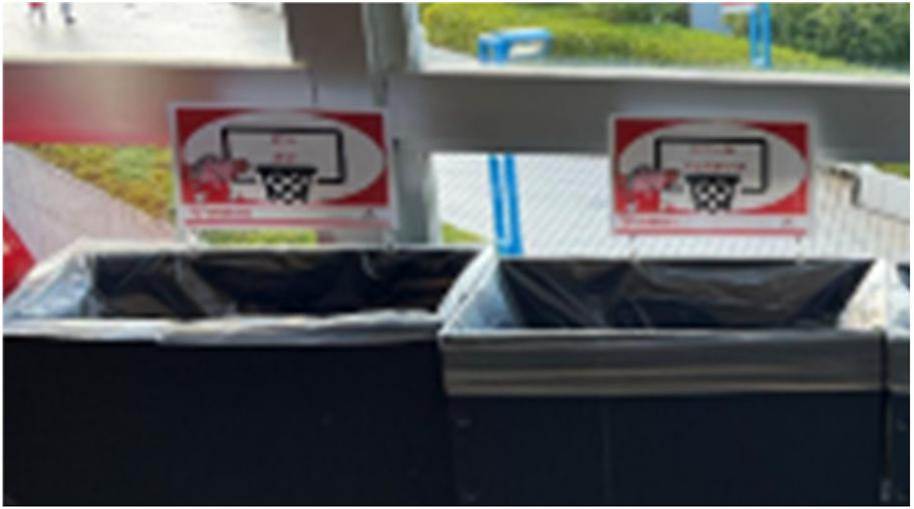 Basketball-style backboard for accurate trash disposal	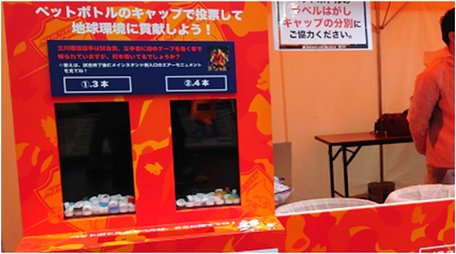 Quiz-style bottle cap voting bin

Envi. denotes environment and AED denotes automated external defibrillator. JEF United denotes JEF United Ichihara Chiba (J. League), CHIBA JETS denotes CHIBA JETS (B. LEAGUE), and Kubota Spears denotes Kubota Spears Funabashi Tokyo Bay (Japan Rugby League One).

On the second day, 243 individuals participated in workshops in which they shared good practices and proposed ideas for improvement for professional sports clubs. The 210 participants of junior high school age or older formed 40 groups and developed 174 improvement proposals for professional sports clubs (Table 7). To examine whether the SMILE program generated improvement proposals that balanced safety and enjoyment, a key design principle of the program, the proposals generated by participants were classified within each SMILE domain according to whether they considered the enjoyment of sports events. As shown in Table 8, improvement proposals that considered the enjoyment of sports events were identified across all SMILE domains. Table 9 presents “mobile staff carrying AEDs” as a specific example of an improvement proposal that considered the balance between safety and enjoyment. Along with a typical HFE-based improvement to enhance the visibility of staff carrying AEDs using fluorescent-colored bibs, a SMILE-based improvement was proposed to align their attire with mascot-themed costume designs to maintain safety while minimizing visual disruption to the event atmosphere.

**Table 7:** Number of workshop participants, groups, and improvement proposals.

Sports club	Date of workshop	Number of participants (Elem.)	Total number of improvement proposals										
			Safe		Move		Info		Link		Envi.		Total
JEF United (Football)	2022/10/22	33 (7)	S1	4	M1	6	I1	5	L1	6	E1	4	25
	2022/10/29	35 (7)	S2	3	M2	9	I2	4	L2	8	E2	8	32
CHIBA JETS (Basketball)	2022/10/29	69 (7)	S3	3	M3	3	I3	2	L3	3	E3	4	38
			S4	6	M4	3	I4	6	L4	5	E4	3	
	2022/11/05	34 (4)	S5	4	M5	4	I5	4	L5	2	E5	3	17
Kubota Spears (Rugby)	2023/01/22	38 (2)	S6	4	M6	5	I6	4	L6	4	E6	6	36
			S7	6	M7	3		-	L7	4		-	
	2023/01/28	34 (6)	S8	5	M8	4	I7	5	L8	2	E7	4	26
				-	M9	2	I8	4		-		-	
Total Mean SD		243 (33) 34.00 15.74		35 4.38 1.19		39 4.33 2.12		34 4.25 1.16		34 4.25 2.05		32 4.57 1.81	174 29.00 7.85

Elem. denotes elementary school students and Envi. denotes environment. JEF United denotes JEF United Ichihara Chiba (J. League), CHIBA JETS denotes CHIBA JETS (B. LEAGUE), and Kubota Spears denotes Kubota Spears Funabashi Tokyo Bay (Japan Rugby League One). S1–S8, M1–M9, I1–I8, L1–L8, and E1–E7 indicate group identifiers within each SMILE domain.

**Table 8:** Examples of improvement proposals that considered the enjoyment of sports events in the SMILE program.

SMILE	Sports club	Representative proposals	Key points for considering event enjoyment
Safe (35)	JEF United (Football) (7)	Allergy information display using QR codes	By utilizing multilingual and visual information displays, the proposal enhances spectators’ sense of security without reducing the enjoyment of watching the event.
	CHIBA JETS (Basketball) (13)	Evacuation route guidance using large monitors	By utilizing existing visual presentation systems in the venue, the proposal supports safe behavior while maintaining spectator immersion.
	Kubota Spears (Rugby) (15)	Mask and AED displays using pop-style signs and color coding	The proposal improves safety without disrupting the spectator experience by presenting alerts in a visually intuitive manner.
Move (39)	JEF United (Football) (15)	Directional guidance using arrows on the floor	By providing intuitive wayfinding, the proposal reduces movement burden and congestion-related stress among spectators.
	CHIBA JETS (Basketball) (10)	Guidance displays using player photographs	By incorporating entertainment elements into venue guidance, the proposal maintains the enjoyable atmosphere of the event even during movement.
	Kubota Spears (Rugby) (14)	Entrance and exit guidance using floor signs	The proposal supports spatial recognition and creates an environment that enables smooth and natural spectator movement.
Info (34)	JEF United (Football) (9)	Player information displays using QR codes	The proposal enhances emotional engagement and participation in spectating by enabling personalized access to information.
	CHIBA JETS (Basketball) (12)	Interactive activities with players and mascots	By reducing the psychological distance between spectators and clubs, the proposal enhances the value of the spectator experience.
	Kubota Spears (Rugby) (13)	QR displays linked to player introductions and videos	By transforming information provision into an entertaining experience, the proposal makes games more enjoyable for novice spectators.
Link (34)	JEF United (Football) (14)	Guidance displays using illustrations and pictograms	The proposal supports inclusive and humorous communication that is easy for children and international spectators to understand.
	CHIBA JETS (Basketball) (10)	Installation of sensory rooms	By creating an environment considerate of noise and crowding, the proposal enables diverse spectators to comfortably enjoy the event.
	Kubota Spears (Rugby) (10)	Character-based trash bins and guidance displays	By presenting guidance and environmental considerations in a friendly manner, the proposal naturally encourages spectator participation.
Envi. (32)	JEF United (Football) (12)	Color-coded and illustrated trash bins	By supporting intuitive and enjoyable waste sorting behavior, the proposal promotes environmental awareness without interfering with the spectator experience.
	CHIBA JETS (Basketball) (10)	SDGs initiatives utilizing waste sorting	By transforming environmental consideration into participatory content, the proposal promotes active spectator involvement.
	Kubota Spears (Rugby) (10)	Environmental displays using mascot characters	By incorporating sports-event-style entertainment elements, the proposal enhances environmental awareness in an enjoyable manner.

Numbers in parentheses indicate the number of improvement proposals. Envi. denotes environment and AED denotes automated external defibrillator. JEF United denotes JEF United Ichihara Chiba (J. League), CHIBA JETS denotes CHIBA JETS (B. LEAGUE), and Kubota Spears denotes Kubota Spears Funabashi Tokyo Bay (Japan Rugby League One).

**Table 9:** Example of balancing safety and enjoyment in sports events: “mobile staff with AED.”

Collected good practice	Typical HFE-based improvement	SMILE-based improvement balancing safety and enjoyment
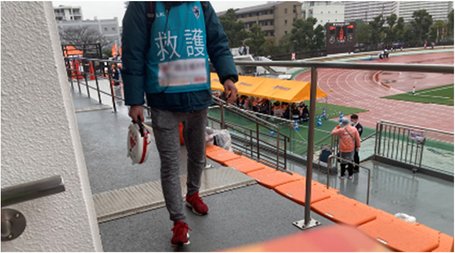 Mobile first-aid staff carrying AEDs and wearing green and white bibs indicating “safe condition” and “first aid” (56,57)	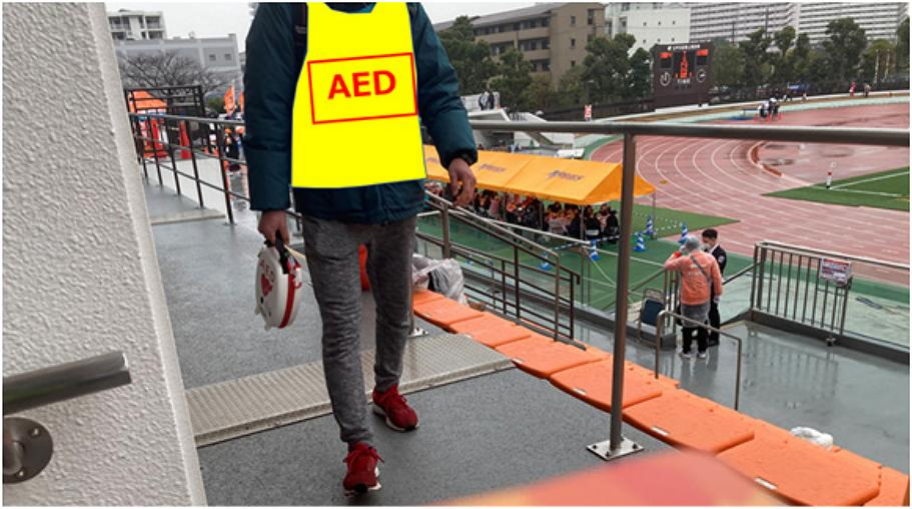 Use of fluorescent-colored bibs to enhance the visibility of mobile first-aid staff carrying AEDs within the venue (58).	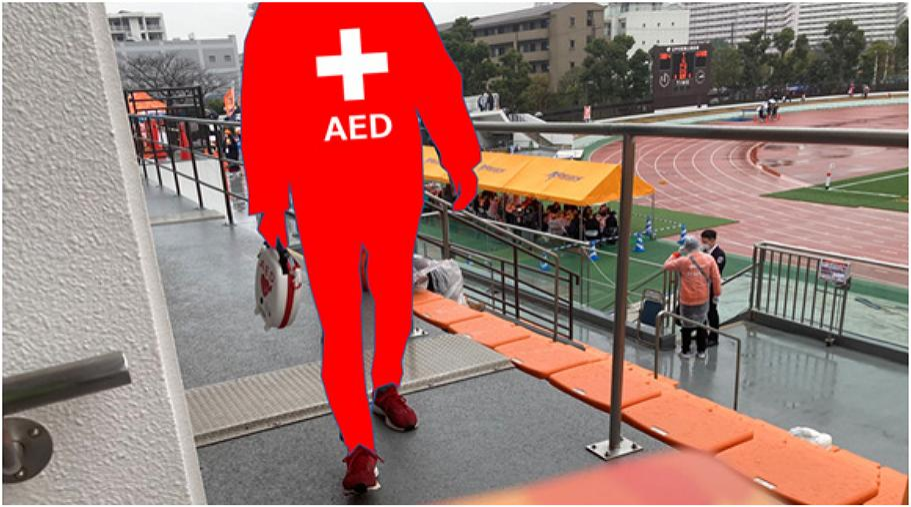 To minimize visual disruption, mobile staff carrying AEDs can align with mascot-themed costume designs for consistency with the event atmosphere.

AED denotes automated external defibrillator.

Regarding program satisfaction, 172 valid responses to the post-test questionnaire were analyzed. The mean overall satisfaction scores, on a 5-point scale, were 4.71 (SD = 0.46) for non-student adults, 4.74 (SD = 0.50) for university students, and 4.88 (SD = 0.35) for junior/senior high school students. One-way ANOVA showed no significant differences in satisfaction scores among non-student adults, university students, and junior/senior high school students for any of the six program components (Table 10). For game observation, the only variable for which Levene's test for homogeneity of variance was significant (*p* = 0.027), Welch's ANOVA was additionally performed and the result remained non-significant (*p* = 0.239). In addition, professional sports clubs provided positive comments regarding the overall implementation of the SMILE program (Table 11).

**Table 10:** Program satisfaction with the draft SMILE program.

	Non-student adults (*n* = 59)	University students (*n* = 105)	Jr./Sr. HS students (*n* = 8)			
Contents	M (SD)	M (SD)	M (SD)	*F*	*p*	*η* ^2^
Fieldwork	4.37 (0.64)	4.57 (0.57)	4.75 (0.46)	2.82	0.063	0.03
Game observation	4.69 (0.50)	4.63 (0.64)	4.88 (0.35)	0.79	0.454	0.01
Introductory session	4.80 (0.41)	4.75 (0.50)	4.75 (0.46)	0.19	0.827	0.00
Group work	4.83 (0.42)	4.82 (0.39)	4.75 (0.46)	0.14	0.868	0.00
Presentations	4.79 (0.49)	4.68 (0.58)	4.75 (0.46)	0.81	0.445	0.01
Overall satisfaction	4.71 (0.46)	4.74 (0.50)	4.88 (0.35)	0.42	0.659	0.01

A one-way analysis of variance (ANOVA) was used to compare the mean scores across three participant categories: non-student adults, university students, and junior/senior (Jr./Sr.) high school students. M (SD) represents the mean and standard deviation. F denotes the F-statistic, *p* denotes the *p*-value, and η^2^ denotes the effect size.

**Table 11:** Representative comments from professional sports clubs regarding the overall implementation of the SMILE program.

Sports club	Representative comment from club staff
JEF United (Football)	We gained many unexpected insights into how spectators perceive the stadium. It was also gratifying to learn that our efforts and intentions behind service provision were recognized by the participants. Although some of the proposed improvements may present practical challenges for implementation, it was meaningful in itself to better understand areas that have not yet been sufficiently addressed.
CHIBA JETS (Basketball)	Previously, we used text-only signs to restrict access, but one participant proposed using a life-size player cutout making a “no entry” gesture instead. We found this idea particularly novel and engaging. It was also reassuring to learn that the intentions behind the services we had provided were being clearly communicated to spectators.
Kubota Spears (Rugby)	Through this program, we became aware of our own unconscious assumptions formed through repeated home games. Even though we believed we were being mindful, we realized we had been thinking, “That's just how rugby games are,” or “We’ve always done it this way.” Hearing fresh opinions from first-time spectators was an invaluable experience, and we believe it will inform our future event operations.

JEF United denotes JEF United Ichihara Chiba (J. League), CHIBA JETS denotes CHIBA JETS (B. LEAGUE), and Kubota Spears denotes Kubota Spears Funabashi Tokyo Bay (Japan Rugby League One).

### 3.4 Results of outcome evaluation (stage 4)

To evaluate the outcomes of the draft SMILE program, we examined pre–post changes in participants’ engagement with the sports club and in spectating sports, supporting sports, participating in sports, and participating in PAOT. To examine potential selection bias, baseline characteristics were compared between the 162 participants with matched pre–post data and the 61 participants who completed the pre-test but did not provide matched post-test data. No significant differences were observed between the two groups in age, participant category, sex, previous viewing experience, or intervention club.

For paired t-tests, the distributions of the pre–post difference scores were assessed based on skewness, kurtosis, and outliers, and no substantial problems with normality were identified. For engagement with the sports club, the scores of pleasure, sign and centrality increased after the program (*d*_z_ = 0.60–0.78) (Table 12). For spectating sports, although the increase in Enjoyment was relatively small, the increases in Awareness, Interest, Opinion formation, and Understanding had small-to-medium effect sizes (*d*_z_ = 0.14–0.73) (Table 12). For supporting sports, all the AEIOU items showed similar increases (*d*_z_ = 0.37–0.66). For participating in sports, only Awareness and Understanding increased slightly (*dz* = 0.25–0.27, respectively). For participating in PAOT, increases were observed for all items, with the largest increase observed for Understanding (*d*_z_ = 0.32–0.84). After the Holm adjustment, the conclusions regarding statistical significance remained unchanged except for Interest in spectating sports, which was no longer statistically significant (Holm-adjusted *p* = 0.136).

**Table 12:** Pre–post comparison of engagement scores before and after the draft SMILE program.

Engagement domain	Pre-test M (SD)	Post-test M (SD)	Mean Difference (95% CI)	*t*	*p*	*d*_z_
(1)Sports club						
Pleasure	10.94 (5.10)	14.07 (4.48)	3.13 (2.42, 3.84)	8.67	<0.001	0.68 (0.51, 0.85)
Sign	9.79 (4.59)	13.03 (4.25)	3.24 (2.60, 3.89)	9.91	<0.001	0.78 (0.60, 0.95)
Centrality	6.13 (3.84)	8.83 (4.56)	2.70 (2.00, 3.40)	7.60	<0.001	0.60 (0.43, 0.76)
(2)Spectating sports						
Awareness	3.77 (0.91)	4.20 (0.82)	0.44 (0.30, 0.58)	6.31	<0.001	0.50 (0.33, 0.66)
Enjoyment	4.25 (0.85)	4.36 (0.84)	0.11 (−0.02, 0.24)	1.73	0.086	0.14 (−0.02, 0.29)
Interest	4.17 (0.88)	4.31 (0.83)	0.14 (0.02, 0.27)	2.23	0.027	0.18 (0.02, 0.33)
Opinion formation	3.25 (1.11)	4.02 (0.92)	0.77 (0.61, 0.94)	9.27	<0.001	0.73 (0.55, 0.90)
Understanding	3.29 (1.07)	3.96 (0.92)	0.67 (0.50, 0.85)	7.51	<0.001	0.59 (0.42, 0.76)
(3)Supporting sports						
Awareness	3.80 (0.98)	4.24 (0.80)	0.44 (0.30, 0.59)	5.90	<0.001	0.46 (0.30, 0.63)
Enjoyment	3.80 (0.96)	4.22 (0.86)	0.42 (0.28, 0.56)	6.10	<0.001	0.48 (0.32, 0.64)
Interest	3.86 (0.99)	4.21 (0.87)	0.35 (0.20, 0.49)	4.71	<0.001	0.37 (0.21, 0.53)
Opinion formation	3.54 (1.12)	4.04 (0.93)	0.49 (0.34, 0.65)	6.22	<0.001	0.49 (0.33, 0.65)
Understanding	3.10 (1.05)	3.75 (0.99)	0.65 (0.50, 0.80)	8.36	<0.001	0.66 (0.49, 0.83)
(4)Participating in sports						
Awareness	3.95 (1.03)	4.19 (0.83)	0.24 (0.10, 0.38)	3.42	<0.001	0.27 (0.11, 0.43)
Enjoyment	4.27 (0.90)	4.33 (0.81)	0.07 (−0.06, 0.19)	1.07	0.284	0.08 (−0.07, 0.24)
Interest	4.22 (0.92)	4.30 (0.86)	0.08 (−0.05, 0.21)	1.21	0.227	0.10 (−0.06, 0.25)
Opinion formation	3.97 (1.12)	4.10 (0.89)	0.13 (−0.01, 0.27)	1.86	0.064	0.15 (−0.01, 0.30)
Understanding	3.69 (1.05)	3.93 (0.92)	0.24 (0.09, 0.38)	3.21	0.002	0.25 (0.10, 0.41)
(5)Participating in PAOT						
Awareness	4.07 (0.89)	4.37 (0.82)	0.30 (0.15, 0.45)	4.01	<0.001	0.32 (0.16, 0.47)
Enjoyment	3.92 (0.89)	4.38 (0.90)	0.46 (0.30, 0.61)	5.76	<0.001	0.45 (0.29, 0.61)
Interest	4.00 (0.91)	4.39 (0.84)	0.39 (0.24, 0.54)	5.26	<0.001	0.41 (0.25, 0.57)
Opinion formation	3.54 (1.06)	4.24 (0.94)	0.70 (0.52, 0.89)	7.63	<0.001	0.60 (0.43, 0.77)
Understanding	3.10 (1.10)	4.07 (0.91)	0.97 (0.79, 1.15)	10.73	<0.001	0.84 (0.66, 1.02)

*n* = 162. (1) Sports club was assessed using PCM items from Kunkel et al. (51): pleasure, sign, and centrality (range = 3–21 each). The score range for each AEIOU item is 1–5. Pre-test M (SD) and post-test M (SD) represent the mean and standard deviation at each time point. 95% CI indicates the 95% confidence interval, t denotes the t-statistic, *p* denotes the *p*-value, and *d*_z_ denotes Cohen's *d*_z_ for paired samples. The Holm adjustment was applied to the pre–post tests across the engagement outcomes; only Interest in spectating sports was no longer statistically significant after adjustment (Holm-adjusted *p* = 0.136).

Finally, ANCOVA was performed to examine whether post-test engagement scores varied according to participant characteristics or external factors after adjusting for baseline scores. In each ANCOVA model, the post-test score was used as the dependent variable; the corresponding pre-test score as the covariate; and participant category (non-student adults, university students, and junior/senior high school students), sex, previous viewing experience, and intervention club as fixed factors. The assumptions for ANCOVA, including residual normality, homogeneity of variance, linearity, and homogeneity of regression slopes, were assessed, and no substantial problems were identified overall.

In the unadjusted analyses, among participant category, sex, previous viewing experience, and intervention club, significant differences in adjusted post-test scores for some items were observed only for participant category. Specifically, these differences were observed for Understanding in spectating sports (F = 4.38, *p* = 0.014, partial *η*^2^ = 0.062), Opinion formation (F = 3.40, *p* = 0.036, partial *η*^2^ = 0.049) and Understanding (F = 3.19, *p* = 0.044, partial *η*^2^ = 0.046) in supporting sports, and Understanding in participating in sports (F = 5.00, *p* = 0.008, partial *η*^2^ = 0.070). However, after applying the Holm adjustment, none of these differences remained statistically significant (Table 13). No significant main effects were observed for sex, previous viewing experience, or intervention club.

**Table 13:** ANCOVA examining factors affecting the interpretation of pre–post engagement scores.

	Adjusted mean (95% CI)					
Engagement domain	Non-student adults (*n* = 59)	University students (*n* = 105)	Jr./Sr. HS students (*n* = 8)	*F*	*p*	partial *η^2^*
(1)Sports club						
Pleasure	14.29 (13.12, 15.45)	14.01 (12.82, 15.21)	16.71 (14.10, 19.32)	2.03	0.136	0.030
Sign	12.77 (11.63, 13.91)	13.10 (11.92, 14.28)	14.01 (11.40, 16.61)	0.49	0.616	0.007
Centrality	8.47 (7.05, 9.89)	8.35 (6.86, 9.84)	10.18 (6.95, 13.41)	0.16	0.856	0.002
(2)Spectating sports						
Awareness	4.17 (3.90, 4.44)	4.27 (3.99, 4.55)	4.30 (3.72, 4.89)	0.50	0.607	0.007
Enjoyment	4.32 (4.06, 4.59)	4.41 (4.13, 4.68)	4.62 (4.04, 5.20)	0.56	0.571	0.008
Interest	4.27 (4.02, 4.52)	4.36 (4.10, 4.63)	4.57 (4.02, 5.12)	0.63	0.533	0.009
Opinion formation	4.05 (3.75, 4.35)	4.16 (3.85, 4.46)	4.33 (3.68, 4.97)	0.81	0.447	0.012
Understanding	3.63 (3.31, 3.95)	4.15 (3.82, 4.47)	4.50 (3.82, 5.18)	4.38	0.014	0.062
(3)Supporting sports						
Awareness	4.08 (3.82, 4.34)	4.18 (3.91, 4.45)	4.18 (3.61, 4.74)	0.78	0.459	0.012
Enjoyment	3.98 (3.72, 4.24)	4.22 (3.95, 4.49)	4.29 (3.72, 4.86)	1.20	0.304	0.018
Interest	4.00 (3.72, 4.27)	4.21 (3.93, 4.50)	4.49 (3.90, 5.09)	1.57	0.212	0.023
Opinion formation	3.77 (3.48, 4.05)	4.14 (3.85, 4.43)	3.81 (3.20, 4.42)	3.40	0.036	0.049
Understanding	3.37 (3.06, 3.67)	3.81 (3.50, 4.13)	3.40 (2.74, 4.05)	3.19	0.044	0.046
(4)Participating in sports						
Awareness	4.21 (3.95, 4.47)	4.13 (3.87, 4.40)	4.42 (3.86, 4.97)	0.20	0.821	0.003
Enjoyment	4.25 (4.01, 4.50)	4.35 (4.09, 4.60)	4.56 (4.03, 5.09)	0.33	0.722	0.005
Interest	4.23 (3.97, 4.49)	4.29 (4.03, 4.55)	4.04 (3.48, 4.60)	1.95	0.146	0.028
Opinion formation	4.04 (3.78, 4.30)	4.07 (3.81, 4.33)	4.11 (3.56, 4.66)	0.14	0.872	0.002
Understanding	3.58 (3.30, 3.86)	4.04 (3.76, 4.32)	3.59 (3.00, 4.18)	5.00	0.008	0.070
(5)Participating in PAOT						
Awareness	4.44 (4.16, 4.72)	4.24 (3.96, 4.52)	4.40 (3.80, 5.00)	0.09	0.910	0.001
Enjoyment	4.48 (4.16, 4.80)	4.24 (3.91, 4.56)	4.50 (3.81, 5.18)	0.28	0.760	0.004
Interest	4.45 (4.17, 4.73)	4.31 (4.02, 4.59)	4.52 (3.92, 5.12)	0.06	0.941	0.001
Opinion formation	4.31 (3.98, 4.64)	4.08 (3.74, 4.42)	3.93 (3.22, 4.64)	0.53	0.589	0.008
Understanding	3.84 (3.53, 4.15)	4.02 (3.70, 4.33)	4.03 (3.38, 4.68)	0.52	0.594	0.008

In the ANCOVA, each post-test score was entered as the dependent variable, the corresponding pre-test score as the covariate, and participant category, sex, previous viewing experience, and intervention club as fixed factors. This table presents the results for participant category. Adjusted means represent estimated marginal means adjusted for the baseline score and the fixed factors included in the model. 95% CI indicates the 95% confidence interval, F denotes the F-statistic, *p* denotes the unadjusted *p*-value, and partial η^2^ denotes the effect size. The Holm adjustment was applied to the tests of the main effects of participant category, sex, previous viewing experience, and intervention club across the engagement outcomes. For participant category, none of the four outcomes with unadjusted *p* < 0.05 remained statistically significant after adjustment: Understanding in spectating sports (Holm-adjusted *p* = 0.315), Opinion formation (Holm-adjusted *p* = 0.761) and Understanding (Holm-adjusted *p* = 0.884) in supporting sports, and Understanding in participating in sports (Holm-adjusted *p* = 0.185).

### 3.5 Results of program refinement (stage 5)

The draft program and checklist were revised based on the results of the pilot. HFE experts, including the facilitators of the pilot sessions, reviewed the draft program from the perspective of efficiency and productivity. They reached a consensus that both the content and timetable of the draft SMILE program (Table 4) were feasible and could be adopted as the refined version without modifications.

Next, the draft action checklist used in the SMILE program was revised. First, the appropriateness of the SMILE domains defined in the draft checklist was examined. Consequently, 95.9% (*n* = 2,831) of the 2,951 good-practice photographs collected during the pilot implementation in Stage 2 were classified into one of the five SMILE domains (S = 360, M = 801, I = 853, L = 406, E = 411) (Table 5). The remaining 4.1% of the photographs (*n* = 120) had initially been categorized as “other”; however, content analysis confirmed that all the photographs could be reclassified into one of the existing SMILE domains. Accordingly, the five SMILE domains defined in the draft checklist were retained as the core structure of the revised action checklist.

The refined checklist items were developed following an inductive content analysis procedure (Table 3). In the open coding stage, 2,951 good-practice photographs classified into SMILE domains were used for inductive content analysis. The two HFE experts jointly reviewed the photographs and conducted open coding, grouping, categorization, and abstraction through discussion, resolving any differences in judgment by consensus. Through this process, six upper-level categories with common meanings were extracted within each SMILE domain. In the abstraction stage, representative good-practice photographs were selected for each category, and corresponding checklist items were developed. All the checklist items in the draft version were replaced with newly developed items derived from the inductive content analysis of the good-practice photographs collected in this study. The wording of the checklist items was revised based on a consensus among the six HFE experts.

Consistent with previous PAOT packages, free-text “blank item” fields were included to allow users to record good practices that did not correspond to predefined domains or checklist items. Each checklist item was accompanied by an illustration based on a representative good-practice photograph of the item.

Consequently, an illustrated action checklist consisting of 30 items (six items for each SMILE domain) and two blank item fields was completed. An example of the refined version of the action checklist is shown in Figure 3. The revised SMILE action checklist, including all 30 items and detailed instructions for its use, is provided in Supplementary Material 2.

**Figure 3:**
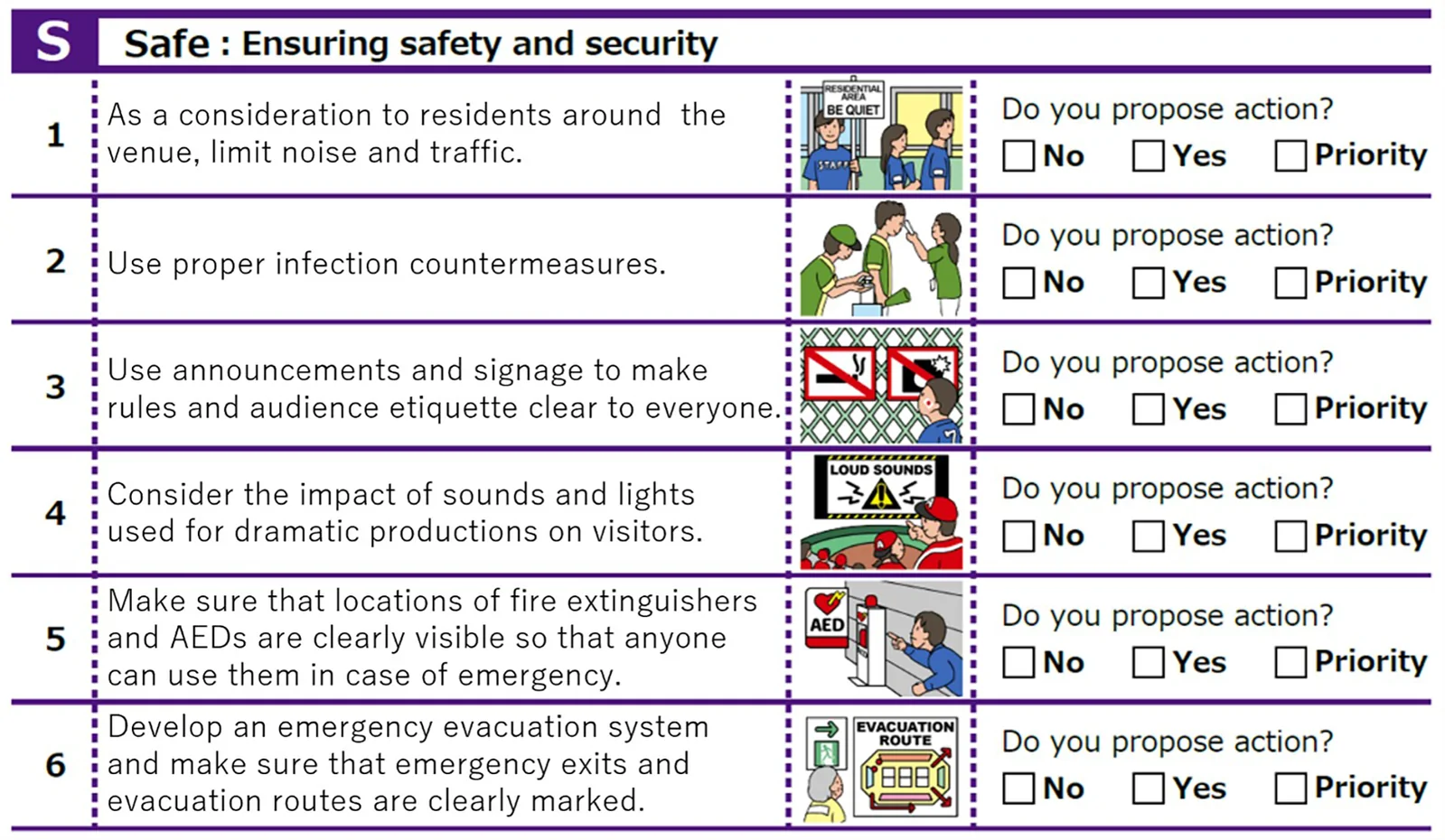
Example of six checkpoints from the “Safe” domain excerpted from the revised 30-item SMILE action checklist. The full 30-item checklist is provided in Supplementary Material 2.

## 4 Discussion

### 4.1 Originality

The originality of this study lies in being, to our knowledge, the first to apply the PAOT framework—originally developed to promote voluntary and sustainable improvements in workplace safety, health, and productivity (28–30)—to sports-event improvement. Unlike workplaces, sports events are often temporally and spatially discontinuous, making it difficult to continuously apply the PDCA cycle and transfer good practices to subsequent events. However, the SMILE program may provide a mechanism for identifying and sharing good practices across sports events. Its potential to facilitate the transfer of good practices across different sports and event contexts requires further evaluation.

Additionally, instead of focusing on problem identification, the SMILE program emphasizes existing good practices and encourages participants to consider improvements in a positive and constructive manner. Thus, a more dialogue-oriented and constructive environment for improving activities can be realized. This contrasts with conventional risk-assessment-based improvement activities, which tend to focus on identifying problems and risks in the field. Because such activities typically foster a blame-oriented atmosphere (59), the SMILE program offers distinct value as a positive, dialogue-oriented alternative for event improvement.

Another originality of the SMILE program lies in its approach to addressing the trade-off between safety and enjoyment. Conventional PAOT packages generally promote improvements aimed at enhancing safety, usability, comfort, and productivity (28, 30, 37, 44–47). However, in sports events, even improvements that are expected to enhance safety may not necessarily be desirable if they reduce spectator immersion or diminish the experiential value of the event. Therefore, rather than treating improvements in safety and enjoyment as separate goals, the SMILE program emphasizes a design philosophy that seeks to achieve improvements while balancing the two. This approach is consistent with previous research highlighting the importance of balancing onsite safety management with the entertainment value of live sports (8).

This design philosophy is illustrated by the improvement proposals generated in this study (Tables 8, 9). Across the five SMILE domains, participants proposed improvements that incorporated sports-event-specific entertainment elements into safety, movement, information, inclusion, and environmental measures (Table 8). The “mobile first-aid staff carrying AEDs” provides a symbolic example of how this design philosophy can be applied (Table 9). The green and white bibs are consistent with ISO 7010 and ISO 3864-1 medical-signage standards (56, 57), supporting their design rationale from a safety perspective. By contrast, under conventional HFE-based improvements, using fluorescent high-visibility bibs is likely to enhance visibility, which is consistent with ISO 20471:2013 standards for high-visibility clothing (58). However, under the SMILE framework, interventions that increase visibility are undesirable if they reduce spectator immersion or lessen experiential value. Instead, improvement proposals, such as using team-colored bibs or adopting designs harmonized with mascot characters, may provide approaches that enhance safety while preserving the experiential value of sports events.

### 4.2 Academic contributions

From an academic perspective, this study contributes to addressing challenges in the field of sports management by applying HFE and PAOT approaches. In particular, an important academic contribution lies in addressing value co-creation in sports events. Although recent literature has increasingly emphasized the concept of value co-creation involving diverse stakeholders (26, 27), systematic methods for facilitating such collaboration in the context of sports spectating have not yet been established. The SMILE program may mitigate this challenge by operationalizing value co-creation through structured workshops (Table 4) and context-sensitive practical tools (Figure 3).

Indeed, a total of 294 participants, including elementary school students, junior and senior high school students, university students, and adult citizens, took part in the fieldwork, of whom 243 participated in the Day 2 workshops (Table 1). Approximately 80% had no previous experience of attending a home game of the professional sports club selected for the program. Nevertheless, participants collected 2,951 photographs of good practices across seven games and generated 174 improvement proposals (Tables 5, 7). These findings suggest that the SMILE program can engage citizens from diverse age groups in identifying and sharing good practices and co-creating further improvements. The comments from the professional sports clubs further indicated that the program provided opportunities to gain new insights from spectators, recognize previously unnoticed assumptions, and identify perspectives that could inform future event operations (Table 11). As demonstrated in this study, citizens may serve not merely as spectators but also as stakeholders who co-create improvements in event environments.

Finally, the program contributes to extending the application of PAOT, which has been developed primarily in the fields of HFE and OSH. From an occupational perspective, sports events can also be viewed as workplaces for staff, athletes, and volunteers, while spectators and citizens participate in the co-creation of services and event environments. This study expands the application of PAOT to sports events, and the SMILE program has already been introduced in international PAOT programs for learning participatory approaches to improving occupational safety and health, including the Mekong Delta and the 2023 and 2025 Japan–Korea PAOT workshops (42, 43).

### 4.3 Practical contributions

From a practical perspective, the program facilitated the co-creation of improvement proposals that considered the balance between safety and enjoyment in sports events through the participation of diverse stakeholders. The program particularly engaged with the “act” stage of the PDCA cycle and provided insights into sustainable and feasible improvements through constructive dialogue among spectators, citizens, club staff, and HFE experts.

The practical significance of the SMILE program lies in its focus on small, low-cost, and immediately actionable improvements that can be identified and adapted to local conditions, thereby supporting sustainable improvement in sports events. Consistent with the good-practice-oriented approach emphasized in PAOT (31, 33), the program did not focus on expensive technological interventions; rather, it aimed to identify feasible improvements already embedded within actual sporting-event environments (Tables 5, 6). Many of the “good practices” reported in the literature on sporting events have focused on mega-sport events and have involved advanced technologies, substantial financial investment, and long-term planning (23–25). However, these approaches are not feasible for all events because they are constrained by event budgets, organizational resources, and venue conditions.

By contrast, the SMILE program was developed based on the assumption that “good practices already exist in the field, even without special or expensive interventions,” reflecting the core principle of PAOT and emphasizing learning from locally existing good practices (31, 32). Using the action checklist, participants can identify good practices observed during sporting events and consider ways to develop feasible improvements based on them (Tables 7, 8). The program also established a participatory process through which locally existing good practices could be continuously identified, shared, and adapted to accumulate small improvements.

Additionally, the findings demonstrate that the SMILE framework was feasible to implement across the three professional sports examined in this study, using both stadium and arena venues. Sports-management studies have suggested that certain common extended elements contribute to spectator sports products, regardless of the sports type or host country (17). Although event operations vary across sports, leagues, and cultural contexts, challenges related to safety, comfort, movement, communication, and environmental considerations may be shared across different spectator sports settings. These characteristics suggest the potential transferability of the SMILE framework to other sporting contexts; however, its applicability beyond the sports, organizations, and regional context examined in this study remains to be evaluated. In addition, because the participants were volunteers recruited in Chiba Prefecture, further evaluation is needed to determine the generalizability of the findings to other participant populations. Practical implementation of the SMILE program also requires prior preparation aligned with the workshop-based PAOT format, including organizer collaboration, adequate workshop facilities, and sufficient participants for forming small groups.

### 4.4 Educational contributions

An important feature of the participants was that approximately 80% had no previous experience of attending a home game of the professional sports club they selected for the SMILE program. Consistent with this limited prior exposure, baseline scores for psychological connection with the sports club were below the midpoint of the possible score range (3–21) for pleasure (10.94), sign (9.79), and centrality (6.13). After participation in the SMILE program, pleasure and sign increased to 14.07 and 13.03, respectively, exceeding the scale midpoint (Table 12). These findings suggest that participation in the SMILE program may provide an opportunity for individuals with limited prior familiarity with a professional sports club to develop a stronger psychological connection with the club.

Furthermore, the observed increases in AEIOU-based engagement with spectating sports, supporting sports, and PAOT suggest that the SMILE program may function as a form of citizen education and science communication (Table 12). For the AEIOU-based engagement measures, most mean scores were in the 3-point range at pre-test, whereas many reached the 4-point range at post-test. Pre–post increases were particularly consistent for spectating sports, supporting sports, and PAOT, whereas changes in engagement with participating in sports were more limited. Given that the AEIOU framework comprises key dimensions of science communication (52, 53), these findings also provide a conceptual basis for interpreting the observed changes as educational and science-communication outcomes. Additionally, these findings are consistent with previous studies that positioned PAOT as a form of science communication in HFE (60). In this context, increased engagement with sports clubs, spectating sports, and supporting sports may reflect learning related to sports management, whereas increased engagement with PAOT may reflect learning related to HFE. Thus, the SMILE program may function as an opportunity for science education that promotes participants’ interest in and learning about both sports management and HFE.

One possible explanation for these observed changes is that the SMILE program follows the workshop-based principles of PAOT and promotes active learning through dialogue, field observations, group work, and proposal-oriented activities (31, 33). Furthermore, dialogue and feedback provided by experts in HFE, OSH, and sports management throughout the workshop may also have contributed to participants’ learning about safety and health and sports management.

### 4.5 Action checklist

The 30-item action checklist was developed through an R&D process involving a pilot implementation and an inductive content analysis of 2,951 photographs of good practices (Supplementary Material 2). The checklist was used to support onsite observations and the development of practical improvement proposals in the context of sports events. The action checklist developed in this study was not intended to function as a psychometric scale requiring standardized validation or reliability testing. Instead, consistent with PAOT principles, it was designed as a practical support tool to identify good practices and develop feasible improvement proposals. PAOT action checklists have been positioned as practical tools for facilitating improvements through participants’ own efforts and promoting sustainable improvement (37). Similar to existing PAOT action checklists, it is intended to be flexibly adapted according to local conditions and should be regarded as a prototype for continued refinement through practice (37, 38, 49).

## 5 Limitations

Although this study involved a relatively large number of participants for a field study and generated substantial empirical data, limitations remain in the research design used to evaluate participant-level outcomes. First, the single-group pre–post comparison design limited the extent to which the observed pre–post changes could be attributed to the SMILE program. The observed changes may also have been influenced by testing effects, the experience of attending a professional sporting event, novelty effects, regression to the mean, or other contextual factors. Second, participants in this study were recruited through volunteer sampling, and participant attrition occurred over the course of the two-day program. Ensuring continued participation in a program requiring participation over two days remains a practical challenge. Third, this study was conducted across seven games involving three professional sports clubs and three sports in one region of Japan. Because participants in the same game shared the same event environment, clustering within games may also have influenced the results. In addition, the findings of this study cannot necessarily be generalized to other sports, clubs, and regions. Finally, while the evaluation based on the AEIOU indicators developed for this study was appropriate for the context of the present study, their validity and reliability could be further strengthened. Future research should accumulate further evidence regarding participant-level outcomes associated with participation in the SMILE program using more rigorous research designs, including control or comparison groups. Furthermore, additional studies should be conducted across different sports, leagues, and regions with diverse cultural backgrounds to enhance the external validity.

## 6 Conclusions

This study developed the SMILE program, a PAOT-based participatory intervention aimed at sustainably improving safety and enjoyment in sports events. The output evaluation confirmed that the program enabled participants to collect good practices that considered the enjoyment of sports events across all SMILE domains and to generate specific improvement proposals, including proposals in the Safe domain that considered the balance between safety and enjoyment. High levels of participant satisfaction were also observed. The outcome evaluation showed that increases in engagement with sports and PAOT were observed following participation in the SMILE program. These findings suggest that the SMILE program may provide a participatory approach that facilitates the sharing and refinement of good practices, the accumulation of small, low-cost, and immediately actionable improvements, and value co-creation among diverse stakeholders involved in sports events.

## Data Availability

Not all photographic data collected at professional sports events can be shared with third parties due to restrictions imposed by the collaborating organizations. Anonymized questionnaire data may be made available by the corresponding author upon reasonable request. Requests to access the datasets should be directed to yayamada@juntendo.ac.jp.

## Supplementary material

Supplementary Material 1 Sports and PAOT engagement items based on the AEIOU model.

Supplementary Material 2 The revised 30-item action checklist for the SMILE program.

## References

[1] Ramchandani G Kokolakakis T Coleman R. Factors influencing the inspirational effect of major sports events on audience sport participation behaviour. World Leis J. (2014) 56:220–35. 10.1080/16078055.2014.938296

[2] Palik M Abdi K. The relationship between watching sport events and spectators’ engagement in physical activities. Pedagog Psychol Med Biol Probl Phys Train Sports. (2018) 22:320–7. 10.15561/18189172.2018.0607

[3] Inoue Y Sato M Filo K Du J Funk DC. Sport spectatorship and life satisfaction: a multicountry investigation. J Sport Manag. (2017) 31:419–32. 10.1123/jsm.2016-0295

[4] Kawaguchi K Ide K Kanamori S Tsuji T Kondo K. Watching sports and happiness among older adults in Japan: the JAGES cohort study. PLoS One. (2025) 20:e0320213. 10.1371/journal.pone.0320213 40202950 PMC11981122

[5] Ramchandani G Coleman R Millar R. The perceived influence of sport event spectatorship on subjective wellbeing. J Glob Sport Manag. (2022) 7:226–41. 10.1080/24704067.2019.1669066

[6] Yoshida M James JD. Customer satisfaction with game and service experiences: antecedents and consequences. J Sport Manag. (2010) 24:338–61. 10.1123/jsm.24.3.338

[7] Ammon R Mahoney K Fried G Arkoubi K Finn D. Roar of the crowd: noise-related safety concerns in sport. J Leg Asp Sport. (2015) 25:10–26. 10.1123/JLAS.2013-0020

[8] Ishii N Nakamura H. Risk management during sport activities: analysis of the recent legal case in Japan regarding foul ball damages on a spectator in a ballpark. Educ Rev USA. (2021) 5:91–7. 10.26855/er.2021.04.002

[9] Block S Lesneskie E. A thematic analysis of spectator violence at sporting events in North America. Deviant Behav. (2018) 39:1140–52. 10.1080/01639625.2017.1409984

[10] DeBenedette V. Spectator violence at sports events: what keeps enthusiastic fans in bounds? Phys Sportsmed. (1988) 16:202–11. 10.1080/00913847.1988.11709465 27404839

[11] Pauser J Schwarz C Morgan J Jantsch J Brem M. SARS-CoV-2 transmission during an indoor professional sporting event. Sci Rep. (2021) 11:20723. 10.1038/s41598-021-99997-0 34671096 PMC8528911

[12] Reade J. Large sporting events and public health and safety. In: Solberg HA Storm R Swart K, editors. Research Handbook on Major Sporting Events. Cheltenham, UK: Edward Elgar Publishing (2024). p. 563–78.

[13] Mason HM King JC Peden AE Leicht AS Franklin RC. The impact of extreme heat on mass-gathering sporting events: implications for Australia and other countries. J Sci Med Sport. (2024) 27:515–24. 10.1016/j.jsams.2024.04.015 38796374

[14] Rahman GR Jasani GN Liang SY. Terrorist attacks against sports venues: emerging trends and characteristics spanning 50 years. Prehosp Disaster Med. (2023) 38:366–70. 10.1017/S1049023X23000377 36938664 PMC10267722

[15] Woodward T Shamim F Hinson M Bass E. Unexpected disasters at organized sporting events. Curr Sports Med Rep. (2015) 14:171–5. 10.1249/JSR.0000000000000163 25968848

[16] Greenwell TC Fink JS Pastore DL. Assessing the influence of the physical sports facility on customer satisfaction within the context of the service experience. Sport Manag Rev. (2002) 5:129–48. 10.1016/S1441-3523(02)70064-8

[17] Mullin BJ Hardy S Sutton WA. The sport product. In: Sport Marketing. 3rd ed. Champaign, IL: Human Kinetics (2007). p. 147–70.

[18] Yoshida M. Consumer experience quality: a review and extension of the sport management literature. Sport Manag Rev. (2017) 20:427–42. 10.1016/j.smr.2017.01.002

[19] Biscaia R Ramos RF Yoshida M Kim Y. Service quality in spectator sports: a review and research agenda. Int J Consum Stud. (2024) 48:e13086. 10.1111/ijcs.13086

[20] Gerrard B. A resource-utilization model of organizational efficiency in professional sports teams. J Sport Manag. (2005) 19:143–69. 10.1123/JSM.19.2.143

[21] Schwarz EC Westerbeek H Liu D Emery P Turner P. Managing Sport Facilities and Major Events. 2nd ed London: Routledge (2016).

[22] Koo GY. Examination of the causal effects between the dimensions of service quality and spectator satisfaction in minor league baseball. Int J Sports Mark Spons. (2009) 11:41–54. 10.1108/ijsms-11-01-2009-b004

[23] AL-Dosari K Deif AM Kucukvar M Onat N Fetais N. Security supply chain using UAVs: validation and development of a UAV-based model for Qatar’s mega sporting events. Drones. (2023) 7:555. 10.3390/drones7090555

[24] Zhu W Feng J Li C Wang H Zhong Y Zhou L. COVID-19 risk assessment for the Tokyo Olympic games. Front Public Health. (2021) 9:730611. 10.3389/fpubh.2021.730611 34760863 PMC8572808

[25] Lusweti S Odawa J. Towards the advanced technology of smart, secure and mobile stadiums: a perspective of FIFA world cup Qatar 2022. Comput Sci Inf Technol. (2023) 11:21–30. 10.13189/csit.2023.110201

[26] Erhardt N Martin-Rios C Chan E. Value co-creation in sport entertainment between internal and external stakeholders. Int J Contemp Hosp Manag. (2019) 31:4192–210. 10.1108/IJCHM-03-2018-0244

[27] Kolyperas D Maglaras G Sparks L. Sport fans’ roles in value co-creation. Eur Sport Manag Q. (2019) 19:201–20. 10.1080/16184742.2018.1505925

[28] International Labour Organization. Participatory Action-Oriented Training (PAOT) (2020). Available online at: https://www.ilo.org/resource/training-material/participatory-action-oriented-training-paot (Accessed August 18, 2026)

[29] Khai TT Kawakami T Kogi K. Participatory Action-Oriented Training. Hanoi: ILO DWT for East and South-East Asia and the Pacific (2011). Available online at: https://www.ilo.org/sites/default/files/wcmsp5/groups/public/%40asia/%40ro-bangkok/%40sro-bangkok/documents/publication/wcms_169357.pdf (Accessed August 18, 2026)

[30] Thurman JE Louzine AE Kogi K. Higher Productivity and a Better Place to Work: Practical Ideas for Owners and Managers of Small and Medium-Sized Industrial Enterprises. Geneva: International Labour Office (1988).

[31] Kogi K. Advances in participatory occupational health aimed at good practices in small enterprises and the informal sector. Ind Health. (2006) 44:31–4. 10.2486/indhealth.44.31 16610530

[32] Kogi K. Practical ways to facilitate ergonomics improvements in occupational health practice. Hum Factors. (2012) 54:890–900. 10.1177/0018720812456204 23397801

[33] Kogi K. Roles of participatory action-oriented programs in promoting safety and health at work. Saf Health Work. (2012) 3:155–65. 10.5491/SHAW.2012.3.3.155 23019528 PMC3443691

[34] Kim SL Lee JE. Development of an intervention to prevent work-related musculoskeletal disorders among hospital nurses based on the participatory approach. Appl Ergon. (2010) 41:454–60. 10.1016/j.apergo.2009.09.007 19875100

[35] Kogi K. Facilitating participatory steps for planning and implementing low-cost improvements in small workplaces. Appl Ergon. (2008) 39:475–81. 10.1016/j.apergo.2008.02.017 18417097

[36] Tsutsumi A Nagami M Yoshikawa T Kogi K Kawakami N. Participatory intervention for workplace improvements on mental health and job performance among blue-collar workers: a cluster randomized controlled trial. J Occup Environ Med. (2009) 51:554–63. 10.1097/JOM.0b013e3181a24d28 19365287

[37] Kawakami T Sano Y. Global Manual for WISE: Work Improvements in Small Enterprises. Geneva: International Labour Organization (2017).

[38] International Labour Organization. Work Improvements in Small Enterprises (WISE): for improving safety and health in plantations (2024). Available online at: https://www.ilo.org/publications/work-improvements-small-enterprises-wise-improving-safety-and-health (Accessed August 18, 2026)

[39] Kogi K. Participatory methods effective for ergonomic workplace improvement. Appl Ergon. (2006) 37:547–54. 10.1016/j.apergo.2006.04.013 16756940

[40] Tsujiura Y Nakao T Kogi K Nguyen TP. Development and application of a participatory training programme on environment education for young people in a rural community in Vietnam. J Sci Labour. (2015) 91:24–38. 10.11355/isljsl.91.24

[41] Tokyo Occupational Safety & Health Center. Work Improvement in Neighbourhood Development for Youth (WINDY) (2020). Available online at: https://mekongdeltapaot.org/windy/ (Accessed August 18, 2026)

[42] Tokyo Occupational Safety & Health Center. The Mekong Delta 2023 report (2024). Available online at: https://mekongdeltapaot.org/2024/02/02/mekong-delta-2023-report/ (Accessed August 18, 2026)

[43] Tokyo Occupational Safety & Health Center. Japan & Korea PAOT workshop (2021). Available online at: https://tokyo-oshc.org/wp/torikumi/global/paot/ (Accessed August 18, 2026)

[44] Hisamune S Kogi K. Findings of the work improvement on board (WIB) programme by the fishery agency in Japan. Int Marit Health. (2015) 66:152–9. 10.5603/IMH.2015.0030 26394315

[45] Kawakami T Kogi K Toyama N Yoshikawa T. Participatory approaches to improving safety and health under trade union initiative–experiences of POSITIVE training program in Asia. Ind Health. (2004) 42:196–206. 10.2486/indhealth.42.196 15128169

[46] Kim S-A Suh C Park M-H Kim K Lee C-K Son B-C. Effectiveness of a comprehensive stress management program to reduce work-related stress in a medium-sized enterprise. Ann Occup Environ Med. (2014) 26:4. 10.1186/2052-4374-26-4 24524591 PMC4026857

[47] Lee JE Kim SL Jung HS Koo JW Woo KH Kim MT. Participatory action oriented training for hospital nurses (PAOTHN) program to prevent musculoskeletal disorders. J Occup Health. (2009) 51:370–6. 10.1539/joh.o8026 19542677

[48] International Ergonomics Association. What is Ergonomics (HFE)? (2000). Available online at: https://iea.cc/about/what-is-ergonomics/ (Accessed August 18, 2026)

[49] International Labour Organization, and International Ergonomics Association. Ergonomic Checkpoints: Practical and Easy-to-Implement Solutions for Improving Safety, Health and Working Conditions. 2nd ed Geneva: International Labour Office (2010). Available online at: http://www.ilo.org/public/libdoc//ilo/2010/110B09_140_engl.pdf (Accessed August 18, 2026)

[50] Písečná M Cihová I. Spectator attendance without spectators: stakeholder insights from a national athletics championships. Front Sports Act Living. (2026) 8:1818383. 10.3389/fspor.2026.1818383 PMC13127306 42065085

[51] Kunkel T Funk D Hill B. Brand architecture, drivers of consumer involvement, and brand loyalty with professional sport leagues and teams. J. Sport Manag. (2013) 27:177–92. 10.1123/jsm.27.3.177

[52] Burns T O'Connor DJ Stocklmayer SM. Science communication: a contemporary definition. Public Underst Sci. (2003) 12:183–202. 10.1177/09636625030122004

[53] Wu LY Wu SP Chang CY. Merging science education into communication: developing and validating a scale for science edu-communication utilizing awareness, enjoyment, interest, opinion formation, and understanding dimensions (SEC-AEIOU). Sustainability. (2019) 11:4551. 10.3390/su11174551

[54] Elo S Kyngäs H. The qualitative content analysis process. J Adv Nurs. (2008) 62:107–15. 10.1111/j.1365-2648.2007.04569.x 18352969

[55] Kim Y-H Yoshikawa E Yoshikawa T Kogi K Jung M-H. Utility of action checklists as a consensus building tool. Ind Health. (2015) 53:85–94. 10.2486/indhealth.2014-0041 25224334 PMC4331198

[56] International Organization for Standardization. ISO 3864-1:2011—graphical Symbols—safety Colours and Safety Signs—part 1: Design Principles for Safety Signs and Safety Markings. Geneva: International Organization for Standardization (2011).

[57] International Organization for Standardization. ISO 7010:2019—graphical Symbols—safety Colours and Safety Signs—registered Safety Signs. Geneva: International Organization for Standardization (2019).

[58] International Organization for Standardization. ISO 20471:2013—high Visibility Clothing—test Methods and Requirements. Geneva: International Organization for Standardization (2013).

[59] Paté-Cornell E Cox LA Jr. Improving risk management: from lame excuses to principled practice. Risk Anal. (2014) 34:1228–39. 10.1111/risa.12241 24989791

[60] Yamada Y Ebara T Toriizuka T. Good practices of ergonomic science communication in Japan. Ind Eng Manag Syst. (2022) 21:670–8. 10.7232/iems.2022.21.4.670

