# Supplementary material for: Development of participatory intervention to improve safety and enjoyment in sports events based on Participatory Action-Oriented Training
Source: Front Sports Act Living. 2026 Oct 23;8:1979235. doi: 10.3389/fspor.2026.1979235 (PMC13741363; doi:10.3389/fspor.2026.1979235)
Supplement: Supplementary file 1 [file Datasheet1.pdf]

Supplementary Material

1 Sports and PAOT engagement items based on the AEIOU model

The items were based on the AEIOU model of science communication (52), with the response format adapted from the SEC-AEIOU questionnaire (53).

1.1 Pre-intervention items

Domain 1. Spectating sports
When you watch sports games, to what extent do the following statements apply to you?
1. I become more aware. (Awareness)
2. I find it enjoyable. (Enjoyment)
3. I am interested in it. (Interest)
4. I have my own opinions and ideas. (Opinion formation)
5. I have sufficient knowledge and understanding. (Understanding)

Domain 2. Supporting sports
When you support the management of sports events, to what extent do the following statements apply to you? Supporting the management of sports events includes not only supporting professional sports events but also sports volunteering, assisting at community sports events, and helping with school sports festivals.
1. I become more aware. (Awareness)
2. I find it enjoyable. (Enjoyment)
3. I am interested in it. (Interest)
4. I have my own opinions and ideas. (Opinion formation)
5. I have sufficient knowledge and understanding. (Understanding)

Domain 3. Participating in sports
When you participate in sports, to what extent do the following statements apply to you?
1. I become more aware. (Awareness)
2. I find it enjoyable. (Enjoyment)
3. I am interested in it. (Interest)
4. I have my own opinions and ideas. (Opinion formation)
5. I have sufficient knowledge and understanding. (Understanding)

Domain 4. Participating in PAOT
Regarding the Participatory Action-Oriented Training (PAOT) for improving sports events that you are about to participate in, to what extent do the following statements apply to you?
1. I become more aware. (Awareness)
2. I find it enjoyable. (Enjoyment)
3. I am interested in it. (Interest)
4. I have my own opinions and ideas. (Opinion formation)
5. I have sufficient knowledge and understanding. (Understanding)

Response scale
1 = Strongly disagree 2 = Disagree 3 = Neither agree nor disagree 4 = Agree 5 = Strongly agree

1.2 Post-intervention items

Domain 1. Spectating sports
When you watch sports games in the future, to what extent do you think the following statements will apply to you?
1. I become more aware. (Awareness)
2. I find it enjoyable. (Enjoyment)
3. I am interested in it. (Interest)
4. I have my own opinions and ideas. (Opinion formation)
5. I have sufficient knowledge and understanding. (Understanding)

Domain 2. Supporting sports
When you support the management of sports events in the future, to what extent do you think the following statements will apply to you? Supporting the management of sports events includes not only supporting professional sports events but also sports volunteering, assisting at community sports events, and helping with school sports festivals.
1. I become more aware. (Awareness)
2. I find it enjoyable. (Enjoyment)
3. I am interested in it. (Interest)
4. I have my own opinions and ideas. (Opinion formation)
5. I have sufficient knowledge and understanding. (Understanding)

Domain 3. Participating in sports
When you participate in sports in the future, to what extent do you think the following statements will apply to you?
1. I become more aware. (Awareness)
2. I find it enjoyable. (Enjoyment)
3. I am interested in it. (Interest)
4. I have my own opinions and ideas. (Opinion formation)
5. I have sufficient knowledge and understanding. (Understanding)

Domain 4. Participating in PAOT
Through participating in the Participatory Action-Oriented Training (PAOT) for improving sports events, to what extent did the following statements apply to you?
1. I become more aware. (Awareness)
2. I find it enjoyable. (Enjoyment)
3. I am interested in it. (Interest)
4. I have my own opinions and ideas. (Opinion formation)
5. I have sufficient knowledge and understanding. (Understanding)

Response scale
1 = Strongly disagree 2 = Disagree 3 = Neither agree nor disagree 4 = Agree 5 = Strongly agree
