# Supplementary material for: Development of participatory intervention to improve safety and enjoyment in sports events based on Participatory Action-Oriented Training
Source: Front Sports Act Living. 2026 Oct 23;8:1979235. doi: 10.3389/fspor.2026.1979235 (PMC13741363; doi:10.3389/fspor.2026.1979235)
Supplement: Supplementary file 2 [file Datasheet2.pdf]

SPORTS SMILE CHECKLIST

Action Checklist for Enjoyable Sporting Events for Everyone

This Action Checklist provides information to ensure the fun, safe, and comfortable operation of sporting events, along with improvements that make the events more enjoyable for everyone involved. Please use it as a reference to improve the environment surrounding events that you are managing, such as sports competitions, community sports meets, and school sports days.

How to use the Sports Smile Checklist

Check the appropriate box under “Do you propose action?” for each item.

1. If a measure is already in place or is not needed, check “No.”
2. If a measure is worthwhile (or improvement is needed), check “Yes.” Even if the measure is already in place, if improvement is needed, check “Yes.”
3. For items checked “Yes,” please check “Priority” for measures that should be prioritized. Three to five measures will be sufficient.
4. Once you complete the checklist, write down “three things that are useful” and “three things that need improvement” to manage fun, safe, and comfortable sporting events that will bring joy to all who are involved (last page).
5. If you have improvement measures that should be added to this checklist, please comment under “Additional Items.”

S Safe : Ensuring safety and security

1	As a consideration to residents around the venue, limit noise and traffic.	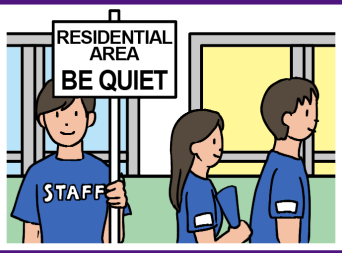	Do you propose action?<br>☐ No ☐ Yes ☐ Priority
2	Use proper infection countermeasures.	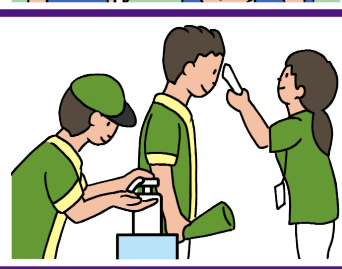	Do you propose action?<br>☐ No ☐ Yes ☐ Priority
3	Use announcements and signage to make rules and audience etiquette clear to everyone.	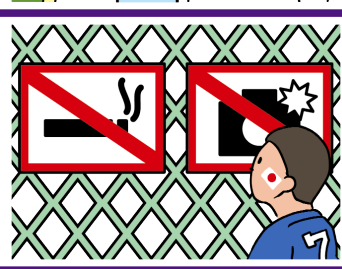	Do you propose action?<br>☐ No ☐ Yes ☐ Priority
4	Consider the impact of sounds and lights used for dramatic productions on visitors.	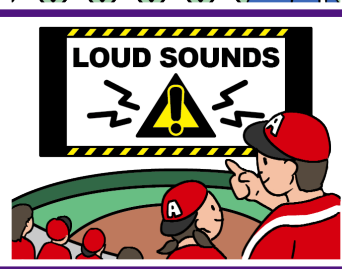	Do you propose action?<br>☐ No ☐ Yes ☐ Priority
5	Make sure that locations of fire extinguishers and AEDs are clearly visible so that anyone can use them in case of emergency.	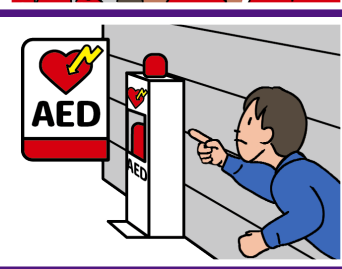	Do you propose action?<br>☐ No ☐ Yes ☐ Priority
6	Develop an emergency evacuation system and make sure that emergency exits and evacuation routes are clearly marked.	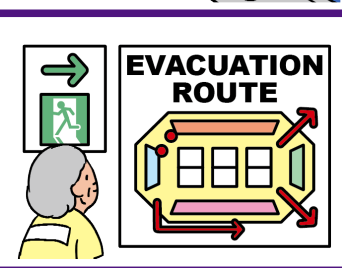	Do you propose action?<br>☐ No ☐ Yes ☐ Priority

M Move : Smooth movement

7	Ensure that visitors do not get lost en route to the venue from transit vehicles and parking lots.	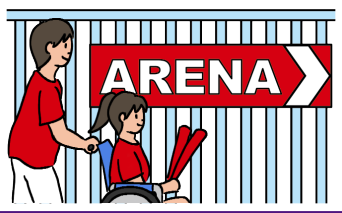	Do you propose action? ☐ No ☐ Yes ☐ Priority
8	Assign staff and place information boards at key points.	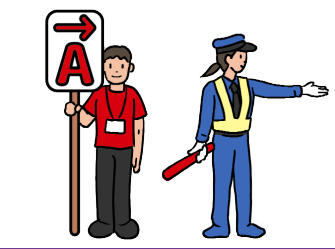	Do you propose action? ☐ No ☐ Yes ☐ Priority
9	Make sure that paths are sufficiently clear to guide the flow of people.	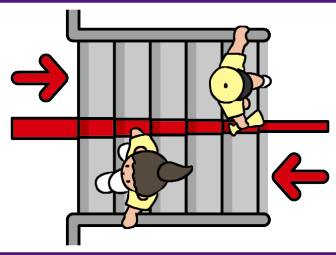	Do you propose action? ☐ No ☐ Yes ☐ Priority
10	Clearly indicate the order of queues and crowded spaces using lines and signage.	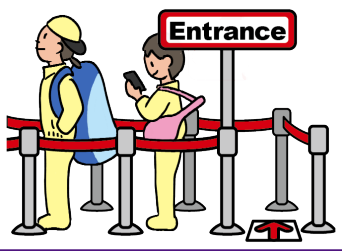	Do you propose action? ☐ No ☐ Yes ☐ Priority
11	Use adequate lighting so that the floor/paths are clearly visible. Protect steps and wires using slopes and covers.	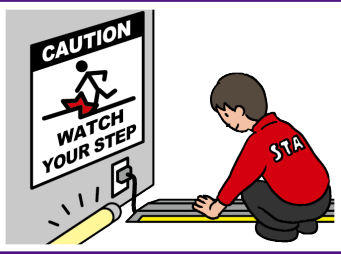	Do you propose action? ☐ No ☐ Yes ☐ Priority
12	Clearly indicate prohibited areas.	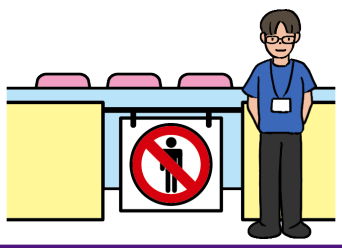	Do you propose action? ☐ No ☐ Yes ☐ Priority

I Info : Communication

13	Share advance information that will be useful for enjoying the event comfortably. Such information will address forecasted weather, event seating, traffic, and parking.	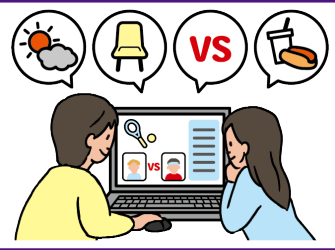	Do you propose action? ☐ No ☐ Yes ☐ Priority
14	Introduce the athletes participating in a sporting event and explain competition rules so that beginners can understand them.	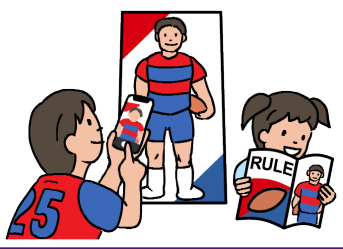	Do you propose action? ☐ No ☐ Yes ☐ Priority
15	Provide time and opportunities for fans to get to know the athletes.	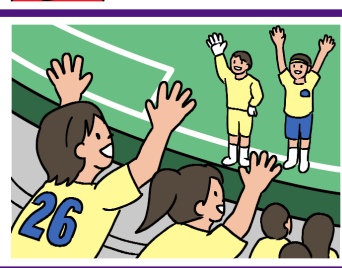	Do you propose action? ☐ No ☐ Yes ☐ Priority
16	Liven up the venue with videos, lighting, sounds, commentaries, et cetera.	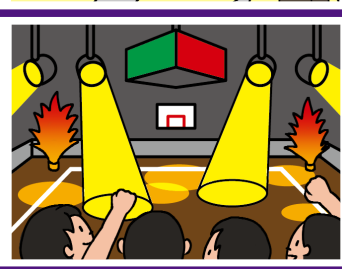	Do you propose action? ☐ No ☐ Yes ☐ Priority
17	Increase the sense of unity in the venue by cheering for the teams, providing novelty goods and team merchandise, and so on.	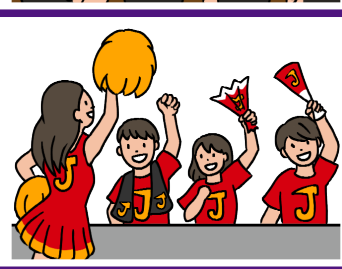	Do you propose action? ☐ No ☐ Yes ☐ Priority
18	Support efforts by community industries, schools, organizations, municipalities, and sponsors.	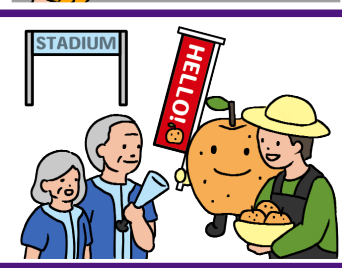	Do you propose action? ☐ No ☐ Yes ☐ Priority

L Link : People-friendly facility maintenance

19	Provide a facilities guide using clear signage, multiple languages, and a variety of colors.	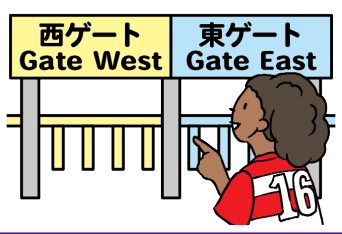	Do you propose action? ☐ No ☐ Yes ☐ Priority
20	Ensure that the venue is bright and that indoor spaces are well-ventilated.	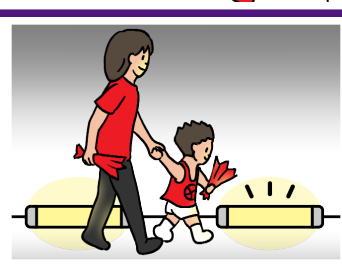	Do you propose action? ☐ No ☐ Yes ☐ Priority
21	Use information and communication technology (ICT), such as digital signage and the Internet, throughout the environment.	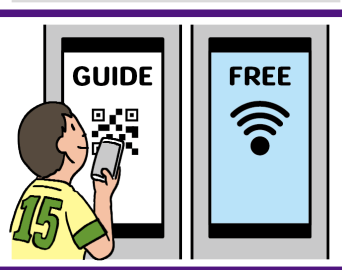	Do you propose action? ☐ No ☐ Yes ☐ Priority

22 Ensure free spaces for visitors to rest comfortably.

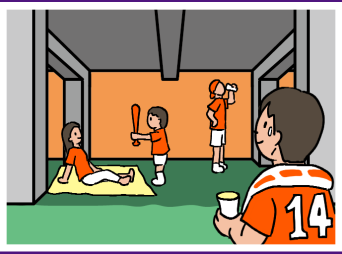

Do you propose action? ☐ No ☐ Yes ☐ Priority

23 Provide a safe place for children to play.

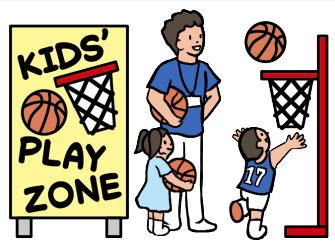

Do you propose action? ☐ No ☐ Yes ☐ Priority

24 Maintain the cleanliness of bathrooms and make them available for everyone.

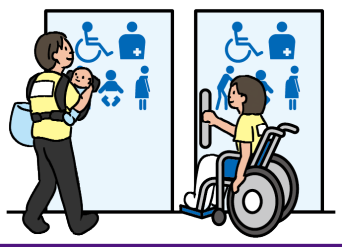

Do you propose action? ☐ No ☐ Yes ☐ Priority

E Environment : Consideration for environmental conservation

25 Create venues and manage events that are environmentally friendly (e.g., avoid the use of plastics).

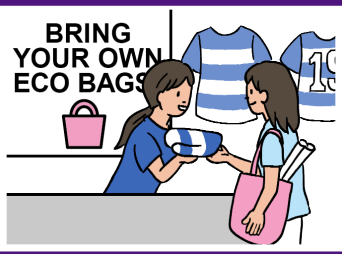

Do you propose action? ☐ No ☐ Yes ☐ Priority

26 Sort garbage using clear signage and colors and promote recycling.

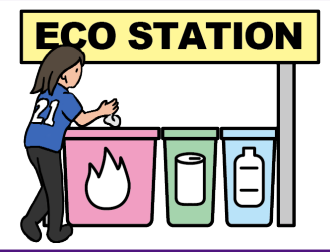

Do you propose action? ☐ No ☐ Yes ☐ Priority

27 Collect garbage at appropriate times according to the timing of competitions and movement of audiences.

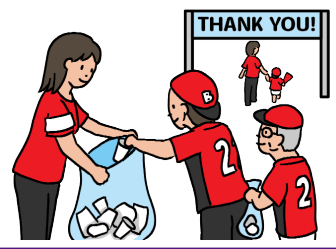

Do you propose action? ☐ No ☐ Yes ☐ Priority

28 Encourage audiences to maintain the cleanliness of competition venues.

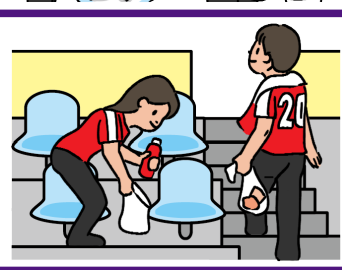

Do you propose action? ☐ No ☐ Yes ☐ Priority

29 Support environmental conservation activities of the community and inform audiences of such efforts.

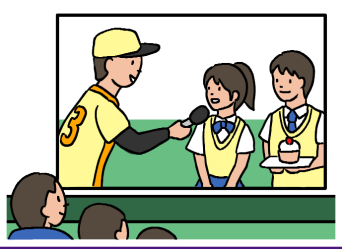

Do you propose action? ☐ No ☐ Yes ☐ Priority

30 Contribute toward a sustainable society with environmental conservation and disaster relief measures.

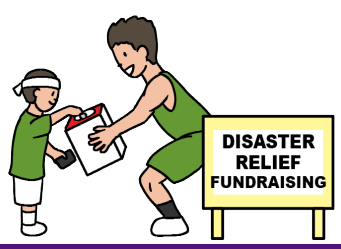

Do you propose action? ☐ No ☐ Yes ☐ Priority

Additional items

31 Please share proposals other than those listed above.

Do you propose action? ☐ No ☐ Yes ☐ Priority

32 Please share proposals other than those listed above.

Do you propose action? ☐ No ☐ Yes ☐ Priority

When the checklist has been completed, please list "three things that are useful" and "three things that need improvement" in order to operate a fun, safe, and comfortable sporting event and bring smiles to all those involved.

Three things that are useful	Three things that need improvement
1	1
2	2
3	3

Prepared by: Juntendo University, "The 2022 Community Vitalization Model Using Varsity Athletics Resources"
(project commissioned by the Japan Sports Agency and Japan Association for University Athletics and Sport)
